# Structural and Dynamic Insights into Podocalyxin–Ezrin Interaction as a Target in Cancer Progression

**DOI:** 10.3390/jox16010025

**Published:** 2026-02-02

**Authors:** Mila Milutinovic, Stuart Lutimba, Mohammed A. Mansour

**Affiliations:** 1 Cancer, Infection and Therapeutics Laboratory, School of Allied Health and Life Sciences, College of Health and Life Sciences, London South Bank University, 103 Borough Road, London SE1 0AA, UK; s4223350@lsbu.ac.uk (M.M.); stuart.lutimba@lsbu.ac.uk (S.L.); 2 Biochemistry Division, Department of Chemistry, Faculty of Science, Tanta University, Tanta 31527, Egypt

**Keywords:** PODXL, Ezrin, R495W mutation, biophysical modelling, cancer metastasis

## Abstract

Cancer metastasis, the spread of tumour cells from the primary site to distant organs, is responsible for over 90% of cancer deaths, yet effective treatments remain elusive due to incomplete understanding of the molecular drivers involved. Podocalyxin (PODXL), a protein overexpressed in many aggressive cancers, links the cell membrane to the internal skeleton through its interaction with Ezrin, an actin cytoskeleton cross-linker. Despite its therapeutic relevance, the PODXL–Ezrin interface remains structurally uncharacterised and pharmacologically intractable. Here, we employed an integrated computational approach combining protein–protein docking, molecular dynamics (MD) simulations, and virtual screening to investigate the structural basis of the PODXL–Ezrin interaction. Using AlphaFold-predicted structures, we modelled PODXL and Ezrin complexes, revealing that PODXL’s cytoplasmic domain stabilises upon Ezrin binding, with Arg495 mediating temporally distinct electrostatic interactions essential for initial complex assembly. Particularly, we characterised the R495W missense mutation in PODXL’s Ezrin-binding domain, demonstrating that substitution of arginine with bulky, hydrophobic tryptophan may allosterically destabilise Ezrin’s dormant conformation. This mutation slightly increases the intramolecular distance between the F3 subdomain and C-terminal domain from 2.59 Å to 3.40 Å, thus leading to potential partial unmasking of the Thr567 phosphorylation site that could plausibly prime Ezrin for activation. Molecular dynamics simulations in the WT state with a total simulation time of 100 ns revealed enhanced structural rigidity and reduced radius of gyration fluctuations in the mutant complex, consistent with a potential “locked,” activation-prone state that amplifies oncogenic signalling. Through virtual screening, we identified NSC305787 as a selective destabiliser of the R495W mutant complex by disrupting key Trp495–pre-C-terminal loop Ezrin interactions and causing steric hindrance to PIP2 recruitment. Our findings identified mutation-dependent changes in drug binding that can guide the development and repurposing of compounds for targeting PODXL-related cancers and improve patient outcomes in PODXL-altered malignancies.

## 1. Introduction

Cancer is one of the leading causes of mortality worldwide, with metastasis accounting for most cancer-related deaths. A deeper understanding of the molecular mechanisms driving oncogenic progression is therefore essential for the development of rational, effective therapies. The Podocalyxin gene, located on chromosome 7q32-33 [1], consists of nine exons which code for a 558-amino acid protein, Podocalyxin (PODXL) [2]. PODXL, a transmembrane sialomucin, is frequently overexpressed in aggressive cancers and is repeatedly linked to poor patient outcomes and invasive behaviour across tumour types [3,4]. It plays a critical role in establishing apical–basal polarity in epithelial, endothelial, and podocyte cells while serving as a scaffold that links the membrane to the cytoskeleton through interactions of its cytoplasmic domain with NHERF1/2 and Ezrin [5,6,7]. In polarised cells, PODXL’s C-terminal PDZ-binding motif (DTHL) binds NHERF1/2, which in turn connects to the Ezrin’s FERM domain, thus driving processes like lumen formation and apical membrane development during 3D tissue morphogenesis [6,7]. The PODXL–NHERF–Ezrin complex dynamically disassembles and reassembles, regulated by PKC- and phosphatase-dependent mechanisms, to control polarity changes and lumen orientation [6]. Furthermore, additional studies report direct interaction between PODXL cytoplasmic tail and Ezrin [8].

During epithelial-to-mesenchymal transition (EMT), PODXL expression is increased, and its direct binding to Ezrin at the juxtamembrane region of cytoplasmic tail promotes dorsal cortical polarity. This polarity facilitates efficient endothelial transmigration and extravasation, key steps in metastasis [9]. Disrupting the PODXL–Ezrin interaction, either genetically or biochemically, prevents these processes, highlighting the complex as a critical driver of metastatic spread. Therefore, targeting the PODXL–Ezrin interaction with small-molecule inhibitors or antibodies could disrupt these pro-metastatic mechanisms, offering a potential therapeutic strategy to limit cancer progression and improve patient outcomes, which forms the main aim of this study.

Ezrin, a 586-amino acid protein encoded by 14 exons on chromosome 6q25.3 [10], is a member of the ERM (Ezrin/Radixin/Moesin) protein family, which cross-links PODXL to the actin cytoskeleton, thereby regulating downstream signalling pathways such as RhoA, Rac1, Cdc42, MAPK-ERK1/2, and PI3K-Akt, that collectively drive cancer cell migration, tumorigenesis, and epithelial–mesenchymal transition [5,11,12,13,14,15]. Based on our previous studies, the mutation of four cytoplasmic lysine residues (K431/525/526/547R; 4K→R) in PODXL leads to a reduction in ubiquitination, resulting in enhanced interaction with Galectin-3 (GAL3), and subsequently increased invasiveness of cells [16]. Therefore, it is plausible that other mutations and post-translational modifications like phosphorylation can also strengthen its interaction with Ezrin and potentiate aggressive cancer phenotypes. Yet the structural underpinnings of the PODXL–Ezrin interaction remain poorly defined, and the impact of specific PODXL mutations on this interface is not fully understood. One of most frequent mutations within the cytoplasmic, Ezrin-binding domain of PODXL, a substitution of Arg495 with Trp495 (R495W) (COSU327, COSU328) [17], warrants further investigation, as its structural consequences on Ezrin binding may provide critical insight into the molecular mechanisms underlying oncogenic signalling.

The absence of high-resolution structural data, such as X-ray crystallography or cryo-EM models, has limited our understanding of the precise binding interfaces, conformational transitions, and dynamic interactions between PODXL’s cytoplasmic tail and Ezrin’s FERM domain [9,18]. This gap is particularly significant for clinically relevant mutations such as one of most prevalent substitutions in PODXL cytoplasmic domain, R495W, whose structural consequences may stabilise or remodel the PODXL–Ezrin complex to potentiate oncogenic signalling [19]. Without such insights, rational strategies for therapeutic disruption of this interaction remain elusive, compounded by the large and dynamic binding surface that has historically hindered drug development [20]. A detailed structural framework is therefore critical not only to identify the molecular determinants of PODXL–Ezrin association but also to reveal how mutations modulate affinity and downstream signalling, opening opportunities for precise therapeutic intervention.

In this study, therefore, we employed an integrated molecular modelling approach combining molecular docking, molecular dynamics simulations, and virtual screening to characterise the structural basis of PODXL–Ezrin interaction. By comparing wild-type and R495W mutant PODXL complexes, we assess how mutation-induced alterations in binding energetics and conformational dynamics shape protein–protein interactions. Furthermore, through in silico screening of small-molecule libraries, we explore potential inhibitors capable of selectively targeting this interface. Importantly, our workflow prioritises the repurposing of compounds, which offers the dual advantage of accelerating clinical translation and reducing development costs. This computational framework not only provides fundamental insights into the biophysical underpinnings of PODXL–Ezrin association but also highlights viable therapeutic avenues for modulating this “undruggable” protein–protein interaction in the context of cancer progression.

## 2. Materials and Methods

### 2.1. Molecular Modelling Tools and Their Applications

This study leveraged a suite of computational tools for simulation, visualisation, and analysis (Table 1). Protein–protein docking was performed using HADDOCK (v2.4) [21,22], an integrative modelling platform, while CASTp (v3.0) was used for the identification of surface pockets, guiding the selection of residues for docking [23]. UCSF Chimera (v1.18) [24] was used for structural visualisation and implementation of the R495W mutation into the PODXL model. Molecular dynamics simulations and subsequent analyses were carried out with OpenMM (v8.1.2) [25] and GROMACS (v2024.2) [26]. CHARMM-GUI (v3.7) [27,28,29] facilitated the generation of simulation-ready systems for both wild-type and mutant PODXL in complex with Ezrin, as well as for ligand-bound complexes, by providing input files and solvation of the system. Interactive visualisation and structural analysis were conducted in PyMOL (v2.5) [30] and VMD (v2.0.0) and the in-house MoLuDock viewer (v1.0) [31]. Ligand–complex receptor docking was accomplished using AutoDock (v4.2.6) [32] against compounds screened through our in-house drug library (MoLuDock lab, London South Bank University) [33], with MGLtools (v1.5.4) [34] for input file preparation and Python (v3.13.3) used for scripting tasks.

### 2.2. Structural Modelling and Optimisation for the PODXL–Ezrin Complex

**Figure 1:**
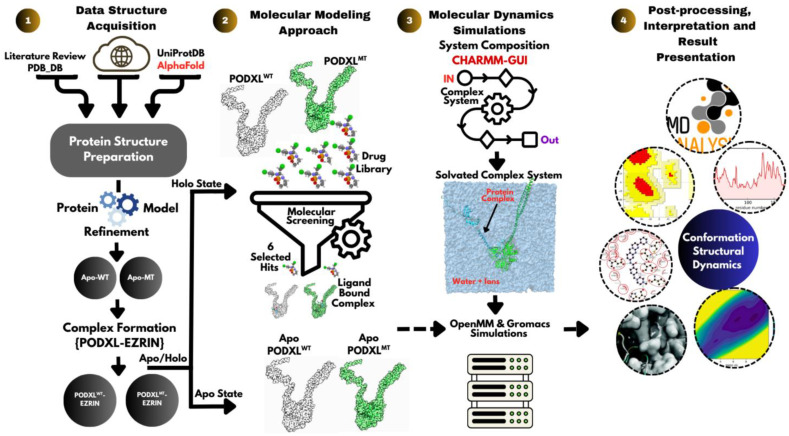
Computational workflow for PODXL–Ezrin interaction analysis and inhibitor identification. Schematic overview of the computational pipeline employed for structural characterisation of PODXL–Ezrin protein complexes and virtual screening of potential therapeutic inhibitors. (**1**) Initial structural models of PODXL and Ezrin proteins were retrieved from protein structure databases and refined using computational modelling approaches. The pathogenic R495W mutation was computationally introduced into PODXL to generate the mutant (MT) protein variant. (**2**) Protein–protein docking algorithms were utilised to assemble PODXL^WT^–Ezrin (grey) and PODXL^R495W^–Ezrin (green) (PODXL^MT^–Ezrin) binary complexes, which subsequently underwent molecular dynamics (MD) simulations to assess structural stability and conformational dynamics. Parallel virtual screening runs were conducted against our in-house drug library to identify putative small-molecule inhibitors targeting the PODXL–Ezrin interface. (**3**) Selected ligand–protein complexes were subjected to rigorous MD simulation protocols implemented in OpenMM, with trajectory analysis performed using the GROMACS analysis toolkit. (**4**) Structural analyses encompassed root mean square deviation (RMSD), root mean square fluctuation (RMSF), inter-residue distance measurements, and radius of gyration (Rg) calculations to elucidate the molecular mechanisms governing PODXL–Ezrin association and to prioritise lead compounds with therapeutic potential as protein–protein interaction inhibitors. Arrows indicate the sequential steps in the computational pipeline.

**Figure 2:**
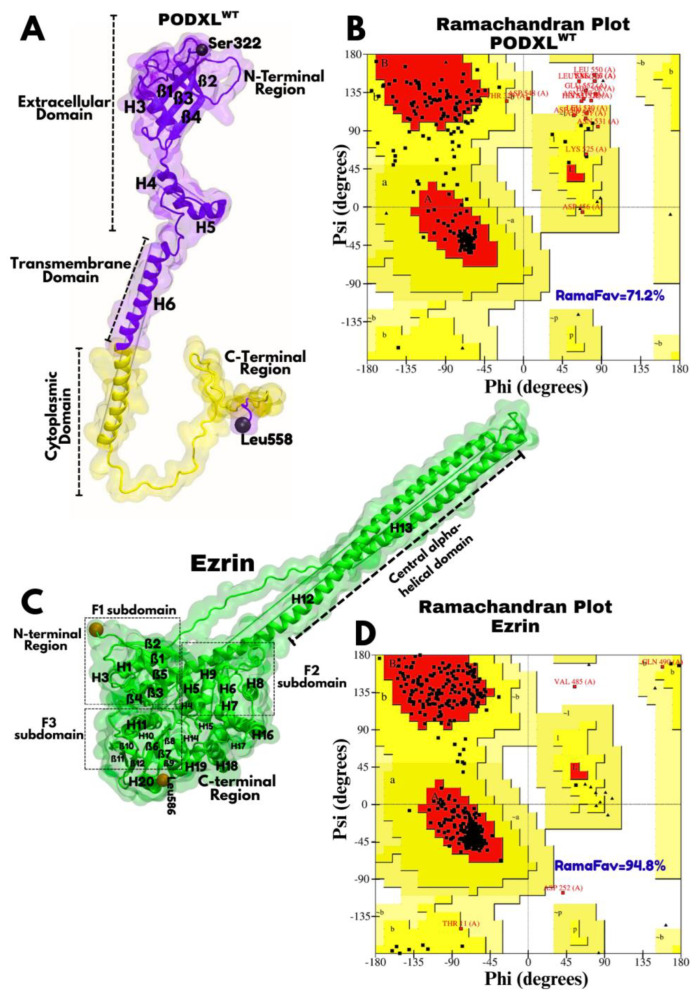
Structural modelling and stereochemical validation of PODXL^WT^ and Ezrin proteins. (**A**) AlphaFold-predicted structure of wild-type human Podocalyxin (PODXL^WT^) spanning residues Ser322 to Leu558. The model highlights the extracellular domain (purple), transmembrane domain (purple), and cytoplasmic domain (yellow), with key secondary structure elements including helices (H3–H6) and beta sheets (β1–β4). Ser322 marks the N-terminal boundary, while Leu558 denotes the C-terminal end, potentially involved in cytoskeletal interactions via adapters like NHERF2. (**B**) Ramachandran plot for PODXL^WT^, showing 71.2% of non-proline and non-glycine residues in the most favoured regions (A, B, L), with outliers (e.g., Leu550, His553) indicating possible flexibility in disordered regions. (**C**) AlphaFold-predicted structure of human Ezrin (green), illustrating the N-terminal FERM domain with F1, F2, and F3 subdomains, the central alpha-helical domain (H12–H23), and the C-terminal actin-binding region. (**D**) Ramachandran plot for Ezrin, with 94.8% of non-proline and non-glycine residues in the most favoured regions, and outliers (e.g., Glu252, Val485) suggesting dynamic or low-confidence regions. Dashed lines in structural models delineate domain boundaries, and Ramachandran plots are colour-coded: red (most favoured), yellow (allowed), with black dots representing residue positions. These data validate the structural integrity of the models, supporting their use in elucidating Podocalyxin–Ezrin interactions in podocyte function and disease contexts. In the Ramachandran plot (**B**,**D**), labels ‘a’, ‘b’, and ‘p’ indicate core/allowed regions for α-helices, β-sheets, and polyproline-like conformations, respectively. Squares (□) mark non-glycine residues, while triangles (△) highlight glycine residues, which can explore broader conformational space.

The three-dimensional structures of full-length PODXL (UniProt ID: O00592; 1846 atoms) and Ezrin (UniProt ID: P15311; 4885 atoms) were modelled with the AlphaFold Protein Structure Database [35,36,37]. Although an X-ray crystal structure of the Ezrin FERM domain is available in the RCSB Protein Data Bank (PDB ID: 1NI2) [38], the predicted full structural model of Ezrin by AlphaFold was selected due to its full-length and high confidence (pLDDT > 80), enabling investigation of the C-terminal actin-binding domain, a region implicated in epithelial–mesenchymal transition (EMT) and cancer metastasis (Figure 1) [9]. At the time of this study (September 2024), no experimentally determined full-length structure of PODXL existed in the PDB, justifying our reliance on the AlphaFold-predicted model (Figure 2).

Evaluation of the PODXL model indicated low prediction confidence (pLDDT < 50) for certain residues in the range of 1–321, suggesting a highly dynamic or disordered N-terminal region with limited structural relevance. To focus on the biologically functional domain and enhance computational tractability, PODXL was truncated to residues 322–558 using VMD. This construct encompassed the cytoplasmic domain (residues 483–558) [39], known to mediate Ezrin binding [5,8], along with a segment of the extracellular and transmembrane regions. This truncation strategy allowed us to study conformational dynamics of PODXL–Ezrin interaction while preserving structural insights for cancer-related processes.

### 2.3. In Silico Mutagenesis of PODXL (R495W Variant with Potential Impact on Ezrin Binding)

**Figure 3:**
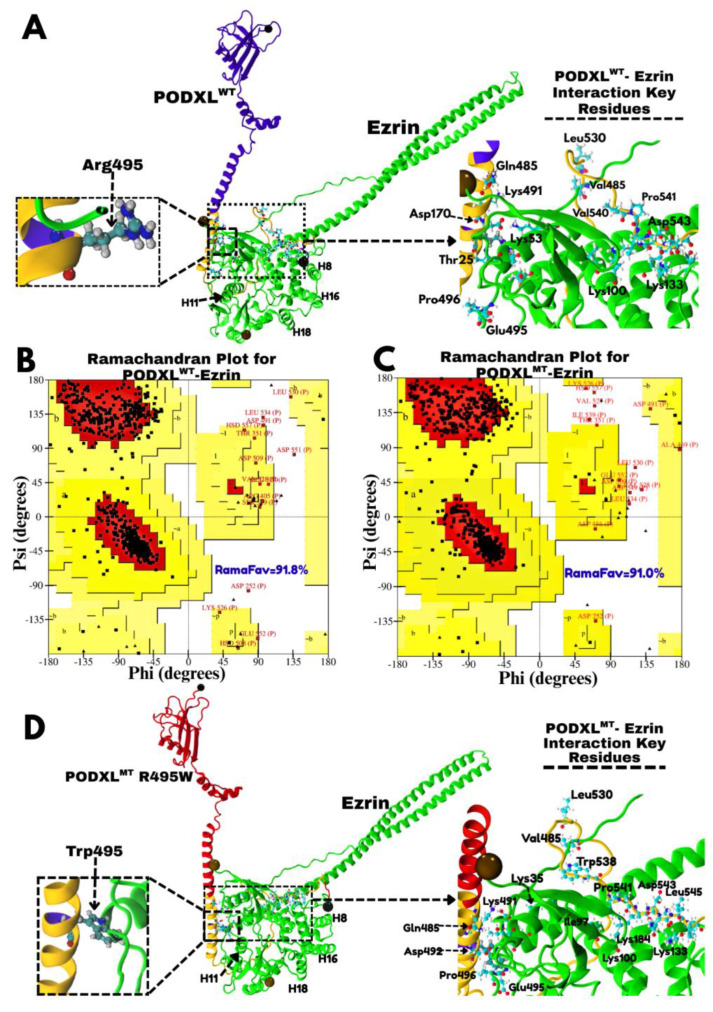
Structural models and stereochemical analysis of PODXL–Ezrin interactions in PODXL^WT^–Ezrin and PODXL^R495W^–Ezrin mutant complexes. (**A**) presents the PODXL^WT^–Ezrin complex (purple for extracellular/transmembrane, yellow for cytoplasmic)–Ezrin (green) complex with a focus on the cytoplasmic–Ezrin FERM domain interface. The zoomed-in box highlights the critical Arg495 residue of PODXL, which participates at the binding interface. To the right, key residues mediating the interaction between PODXL^WT^ and Ezrin are displayed as stick models. (**B**) Ramachandran plot for the PODXL^WT^–Ezrin complex, showing the distribution of backbone dihedral angles (ϕ,ψ). Most residues (91.8%) are in the favourable regions; outliers and their residue names are annotated, reflecting good stereochemical quality for the modelled structure. (**C**) Ramachandran plot for the PODXL^MT^–Ezrin (R495W mutant) complex. Similarly, 91.0% of residues are in favoured regions, and noted outliers are indicated. This plot compares the overall conformational quality with the wild-type model complex. (**D**) Structural model of the PODXL^R495W^–Ezrin mutant. PODXL (red) in complex with Ezrin (green), with zoom by the arrow showing the substitution of arginine to tryptophan at position 495 (Trp495). The interface residues identified as key for the interaction in the mutant context are visualised, illustrating mutation-induced structural alterations at the binding site. In Ramachandran plots (**B**,**C**), ‘a’, ‘b’, and ‘p’ label the core/allowed regions for α-helices, β-sheets, and polyproline-like conformations, respectively. Squares (□) represent non-glycine residues; triangles (△) indicate glycine residues, which occupy a wider conformational space.

The R495W missense mutation was introduced into the PODXL structure using the Rotamers tool in UCSF Chimera. This substitution was selected based on our findings identifying Arg495 as a critical residue at the PODXL–Ezrin interface (see Section 3.4). Furthermore, the R495W variant is among the most frequent amino acid substitutions within the cytoplasmic, Ezrin-binding region of PODXL (residues 483–558), as presented by the COSMIC database [17], with two documented occurrences in pancreatic tissue samples (COSU327, COSU328) [40]. UCSF Chimera was utilised for mutagenesis due to its capacity to evaluate rotameric states based on probability and optimise side-chain conformation within the structural environment. The most presented rotamer for Trp495 was selected from the Dunbrack 2010 library, with consideration of hydrogen bonding and steric compatibility (Figure 3D). This rational mutagenesis approach enabled a systematic assessment of the structural and functional consequences of the R495W mutation on the PODXL–Ezrin interaction, providing insight into its potential role in cancer pathogenesis.

### 2.4. Wild-Type and R495W PODXL–Ezrin Complex Modelling and Stereochemical Validation

The protein–protein interactions between Ezrin and both wild-type PODXL (PODXL^WT^) and mutant (PODXL^R495W^) were modelled and compared using the HADDOCK web server. HADDOCK is an integrative modelling platform that generates high-quality biomolecular complexes by incorporating biochemical data and structural information [21]. Based on prior evidence indicating a direct association between the Ezrin FERM domain and the cytoplasmic region of PODXL [8], residues 483–554 of PODXL [39] were defined as active residues to guide the docking. This selection was further supported by studies showing that deletion of the cytosolic tail beyond residue 452 abrogates PODXL-mediated adhesion [41]. Although the C-terminal DTHL motif (residues 555–558) of PODXL is critical for binding PDZ-domain proteins such as NHERF2 [7], it was omitted from the active residue set as it is not essential for Ezrin binding [8]. We used a full-length Moesin structure from *Spodoptera frugiperda* on RCSB PDB (PDB ID: 2I1K) as a template model for identifying surface binding sites via CASTp, guiding our selection of active residues in protein–protein docking. This was conducted due to the lack of fully resolved crystallographic structure of Ezrin and the high sequence similarity of Moesin [*Spodoptera frugiperda*] and Ezrin [*Homo sapiens*] FERM domains (79%, RMSD = 1.21). This resulted in a selection of 40 residues predominantly located in F1 and F2 subdomains (Figure S1A) at which 70% was identical between Moesin and Ezrin, further supporting our choice. Despite CASTp prediction for the Ezrin FERM domain (PDB ID: 1NI2) involving residues in the F3 subdomain as well, we omitted including them in our selection. Firstly, although not completely buried, residues in the F3 subdomain are known to be interacting with the C-terminal domain in the dormant state of Ezrin, used in our analysis [42], as discussed in Section 3.1. Secondly, K253 and K254, reported on CASTp, are known binding sites of phosphatidylinositol 4,5-bisphosphate (PIP2), amongst K262 and K263 [43], and therefore were excluded from analysis of the interaction with PODXL specifically. This data-driven approach ensures our docking simulations targeted a biologically relevant interface.

HADDOCK improves docking reliability by randomly excluding 50% of the ambiguous interaction restraints (active and passive residues) in each run to mitigate potential false positives [44]. The server was configured to automatically remove buried active residues and define surrounding passive residues. Complexes were clustered using a Fraction of Common Contacts (FCCs) cutoff of 0.60. For the PODXL^WT^–Ezrin complex, 114 structures were grouped into 11 clusters, with Cluster 1 (HADDOCK score: −13.8 ± 7.0) identified as the most reliable. For the PODXL^R495W^–Ezrin complex, 121 structures formed 9 clusters, with Cluster 1 (HADDOCK score: −0.4 ± 7.3) selected. Stereochemical quality of the final docked complexes, along with individual protein models, was assessed using PROCHECK (v3.5) [45,46] with a resolution parameter of 1.5 Å. Ramachandran plots (Figure 2 and Figure 3) were generated to evaluate backbone dihedral angles (φ and ψ), classifying residues into favoured, allowed, and disallowed regions [47]. This validation step confirmed the absence of steric clashes and improper bond geometry, ensuring structural integrity for subsequent molecular dynamics simulations and downstream analyses.

### 2.5. System Preparation and Molecular Dynamics Simulations of PODXL^WT^–Ezrin and PODXL^R495W^–Ezrin Complexes

The PODXL^WT^–Ezrin and PODXL^R495W^–Ezrin complexes were prepared and assembled for molecular dynamics (MD) simulations using CHARMM-GUI. Each complex was solvated in a cubic water box with a 10 Å buffer using the TIP3P water model [48] under periodic boundary conditions. Box dimensions were 214 × 214 × 214 Å^3^ for the WT complex system and 219 × 219 × 219 Å^3^ for the R495W mutant system. To mimic physiological conditions, sodium and chloride ions were added to a concentration of 0.15 M, resulting in 889 Na^+^ and 867 Cl^−^ ions for the WT system and 953 Na^+^ and 930 Cl^−^ ions for the mutant system. Following solvation and ionisation, the systems comprised 928,620 atoms (304,474 water molecules) for the WT complex and 994,954 atoms (326,543 water molecules) for the R495W complex. To model the potential energy of the molecular systems, the CHARMM36m force field [49] was used for improved accuracy for modelling protein dynamics. Given that PODXL–Ezrin binding occurs in the cytoplasm, an aqueous solvated system was selected to best approximate the physiological environment.

MD simulations were performed using GROMACS and OpenMM. GROMACS was chosen for its computational efficiency and integrated analysis tools, while OpenMM provided flexibility for customising simulation parameters via Python scripting. Both protein state systems underwent energy minimisation using the steepest descent algorithm (2000 steps), followed by equilibration in the NVT ensemble with a Nosé–Hoover thermostat (310.15 K) and the NPT ensemble with a Martyna–Tobias–Klein barostat (1 atm). MD simulation runs were conducted for 100 ns and 20 ns with a 2 fs time step for PODXL^WT^–Ezrin and PODXL^R495W^–Ezrin complexes, respectively. A simulation time of 20 ns for the mutant complex was deemed sufficient based on RMSD convergence, indicating that structural equilibrium was reached. While extending the simulation may provide deeper insights, this time frame was considered adequate to capture a glimpse of the system’s dynamics. Nevertheless, all subsequent analyses were performed using the initial 20 ns of the wild-type complex simulation, and the complete 20 ns of the mutant one, allowing for consistent comparison between the systems.

### 2.6. Structural Analysis and Visualisation of Docking and MD Results

Following molecular docking and dynamics simulations, structural analysis and visualisation were performed using UCSF Chimera, PyMOL, and VMD. VMD was used to visualise the simulation trajectories by loading the combined trajectory (DCD) and parameter (PSF) files. To assess conformational stability, root mean square deviation (RMSD) and root mean square fluctuation (RMSF) were calculated using an in-house script. The resulting values were plotted as a function of simulation time (20 ns) using XMGrace (v5.1.22), enabling comparative analysis of the wild-type and R495W PODXL–Ezrin complexes. Inter-residue distances within the binding interface were monitored using a cutoff of 3 Å, applied to atom pairs identified in the initial simulation frame (frame 0). Distance profiles were also visualised in XMGrace to track the evolution of key interactions throughout the trajectory of 20 ns.

### 2.7. Virtual Screening Workflow for Inhibitor Identification

An in-house computational drug library (MoLuDock lab, London South Bank University) comprising over two thousand compounds curated from PubChem [50] was prepared for virtual screening. Compounds were selected based on their potential to disrupt the PODXL–Ezrin interaction. Using Open Babel (v3.1.0) [51], canonical SMILES strings were converted to 3D structures in PDB format, protonated at pH 7.0, and optimised by adding polar hydrogens and partial charges to ensure docking compatibility. Molecular docking was performed with AutoDock, which employs an empirical scoring function and a Lamarckian genetic algorithm to predict ligand binding modes and affinities [52]. Receptor and ligand structures were prepared in PDBQT format, incorporating polar hydrogens and Kollman charges. Using AutoDock Vina (v1.2.0) [53,54], the grid box was centred on the PODXL–Ezrin interface (centre: x = −23.188, y = −16.068, z = −14.338; dimensions: 122 × 126 × 126 Å^3^; spacing: 0.375 Å) to confine the search space and improve computational efficiency. Docking exhaustiveness was set to 10, and the energy range was maintained at 3.0 kcal/mol. The resulting poses were visualised in PyMOL, and the conformation with the lowest binding energy was selected for each ligand among the two studies’ complex states.

### 2.8. MD Simulations and Analysis of Ligand-Bound Complexes

The top-scoring ligand–receptor complexes were subjected to molecular dynamics simulations using the same protocol as for the apo systems. Each complex was solvated in a TIP3P water box with 0.15 M NaCl, using CHARMM-GUI and the CHARMM36m force field. Ligand parameters were generated via the CGenFF tool (v5.0). Energy minimisation (steepest descent, 2000 steps) was followed by NVT and NPT equilibration at 310.15 K and 1 atm. Production MD runs were conducted for 20 ns using GROMACS and OpenMM. Due to system size, water and ions were excluded prior to analysis using VMD, resulting in ~13,500–13,600 atoms per system. Trajectories were analysed using MDAnalysis in Python to compute RMSD, RMSF, radius of gyration, and centre of mass (CoM) distances. This integrative approach enabled the comparative assessment of complex stability and ligand efficacy, identifying promising candidates for experimental validation.

## 3. Results

### 3.1. Structural Modelling and Validation of PODXL and Ezrin

The structural model of PODXL (residues 322–558), modelled from the AlphaFold Protein Structure Database (UniProt ID: O00592), comprises an extracellular domain (Figure 2A), a transmembrane helix presented in purple, and a cytoplasmic domain terminated at Leu558 presented in yellow (Figure 2A). The extracellular domain (residues 322–461) adopts a compact fold characterised by a combination of beta sheets (β1, β2, β3, β4) and alpha helices (H3, H4, H5), forming a globular structure that likely corresponds to the cysteine-rich region conserved among CD34 family members [55]. This region is predicted with moderate to high confidence (90 > pLDDT > 70), suggesting a stable core that may facilitate interactions with extracellular ligands or glycosylation sites critical for Podocalyxin’s anti-adhesive properties in podocytes and endothelial cells. Transitioning into the transmembrane domain (residues 462–482, Figure 2A), the structure features a consistent helical topology expected for single pass transmembrane proteins. The cytoplasmic domain (residues 483–558), depicted in a distinct conformation, assumes a predominantly coiled and extended structure with minimal secondary elements, terminating at Leu558. The observed tail is known to interact with intracellular partners such as NHERF2 and Ezrin, linking Podocalyxin to the actin cytoskeleton, which is essential for maintaining podocyte foot process integrity and preventing proteinuria in glomerular function [7]. However, the low-confidence regions in the cytoplasmic tail (pLDDT < 50) may indicate intrinsic disorder.

The Ezrin model (UniProt ID: P15311) consists of an N-terminal FERM domain containing F1 (residues 2–82), F2 (96–198), and F3 (204–296) subdomains, a central alpha-helical domain (H12 (residues 326–406) and H13 (residues 417–466)), and a C-terminal actin-binding region (residues 497–586) (Figure 2C) with high confidence in the predicted fold (pLDDT scores > 80). In our study, we refer to the region between central alpha-helical and C-terminal domain as the pre-C-terminal loop (residues 467–496). Residues within the F1 and F2 subdomains (highlighted in orange, Figure 2C) defined the docking boundary as inferred from CASTp analysis. The FERM domain extends into a prominent central alpha-helical domain. This elongated helical region, depicted with high structural confidence, is indicative of the coiled-coil or stalk-like structure typical of ERM proteins in their dormant state, where the N-terminal, specifically the F3 subdomain, and C-terminal domains are thought to interact intramolecularly [42,56]. Activation of Ezrin, on the other hand, is a result of various processes, including binding with phosphatidylinositol 4,5-bisphosphate (PIP2) followed by phosphorylation of Thr567 [57]. Phosphorylation of Thr567 is thought to open the mentioned N-C-terminal binding, thereby transforming Ezrin into an active state [58]. Despite the availability of only the inactive form on AlphaFold (UniProt ID: P15311), our selection of residues, followed by MD simulations, provides relevant insight into the association between Podocalyxin and Ezrin.

Stereochemical quality was assessed using Ramachandran plots, excluding proline and glycine residues due to their atypical stereochemistries [59]. The unbound Ezrin model exhibited high structural quality, with 94.8% of residues in the most favoured regions (Figure 2D). In contrast, the unbound PODXL model showed greater flexibility, with only 71.2% of residues in favoured regions (Figure 2B). However, upon complex formation with Ezrin, the percentage of favoured residues in PODXL increased to 91.8% (Figure 3B), indicating that the initial lower value likely reflects intrinsic flexibility in the apo state rather than poor model quality. This improvement supports the physiological relevance of the docked complex.

### 3.2. Structural Insights into PODXL^WT^–Ezrin Interactions and the Impact of the R495W Mutation on Complex Stability

Protein–protein docking using HADDOCK 2.4 revealed a stable interaction between the cytoplasmic domain of Podocalyxin (PODXL) and the FERM domain of Ezrin, consistent with prior experimental evidence demonstrating that this association modulates cytoskeletal dynamics and enhances cancer cell aggressiveness. Representative model structures from HADDOCK 2.4 clusters were selected to analyse key interactions and structural dynamics changes. The PODXL^WT^–Ezrin complex (Figure 3A) comprises 823 residues, with the PODXL cytoplasmic tail (highlighted in yellow) forming a close interface with Ezrin’s FERM domain in green. This configuration positions the extracellular and transmembrane domains of PODXL, as well as the central alpha-helical and C-terminal actin-binding domains of Ezrin, in orientations that remain accessible for independent functions, such as extracellular ligand binding or actin cytoskeleton linkage. Key interaction residue at the interface includes Arg495 on PODXL, which forms potential hydrogen bonds or electrostatic interactions with Ezrin’s residues. PODXL is observed to predominantly interact by its cytoplasmic domain coloured in yellow with Ezrin featuring a network of interacting amino acids in the stability of the complex, as visualised in the zoomed insert.

Stereochemical validation via Ramachandran plots confirmed the high quality of the docked models. For the PODXL^WT^–Ezrin complex (Figure 3B), 91.8% of non-proline and non-glycine residues occupy the most favoured regions, with minimal outliers (e.g., Leu530, Asp551, Asp252) likely corresponding to flexible loops at the periphery of the interface. Similarly, the PODXL^R495W^–Ezrin mutant complex (Figure 3C) exhibits 91.0% residues in favoured regions, indicating comparable structural integrity despite the mutation, though outliers like Val527, His557, and Thr351 suggest subtle conformational adjustments in the binding site. As discussed in Section 3.4, Arg495 stands out as a residue in PODXL which engages in two distinct interactions with residues within Ezrin, suggesting its dual, yet functionally significant role in this complex. Furthermore, as per the COSMIC database, substitution of Arg495 with Trp495 is among the most prevalent mutations located in the cytoplasmic, Ezrin-binding region of PODXL (COSU327, COSU328) [17]. These observations motivated further analysis of both the wild-type and mutant complexes, to assess the structural and functional implications of the R495W mutation.

In the PODXL^R495W^–Ezrin mutant complex (Figure 3D), the substitution of Arg495 with Trp495 introduces a bulkier, hydrophobic side chain, potentially altering the interaction landscape. The model shows Trp495 oriented toward Ezrin residues such as Asp492, which may weaken electrostatic interactions while introducing pi-stacking or hydrophobic contacts. This structural shift complements existing studies on PODXL variants in cancer, where mutations in the Ezrin-binding region, as catalogued in COSMIC, correlate with pancreatic tumorigenesis (COSU327, COSU328) [17,40].

In addition, to quantitatively assess the mutation’s effect on Ezrin’s conformational state, we measured the distance between Pro236^HD1^ (F3 subdomain) and Thr567^HG21^ (C-terminal), a representative intramolecular interaction between residues located in domains (F3 and C-terminal) that maintain Ezrin’s dormant form. In the wild-type complex, this distance was 2.59 Å in the last frame of 20 ns simulation, which is consistent with a closed, inactive state (Figure S2B). Strikingly, in the R495W mutant complex, this distance increased to 3.40 Å at the end of simulation, suggesting an early destabilisation event which may precede unmasking of Thr567 for phosphorylation and priming Ezrin for activation (Figure S2C). Pairwise structural alignment (TM-align method) further confirmed a significant rearrangement in the F3-C-terminal domains between the wild-type and mutant complexes (RMSD = 2.12 Å; Figure S2A). These findings indicate that the R495W mutation allosterically slightly destabilises Ezrin’s dormant conformation, potentially shifting it toward an active state conducive to oncogenic signalling.

### 3.3. Structural Dynamics and Mutation-Induced Stability of the PODXL–Ezrin Complex

**Figure 4:**
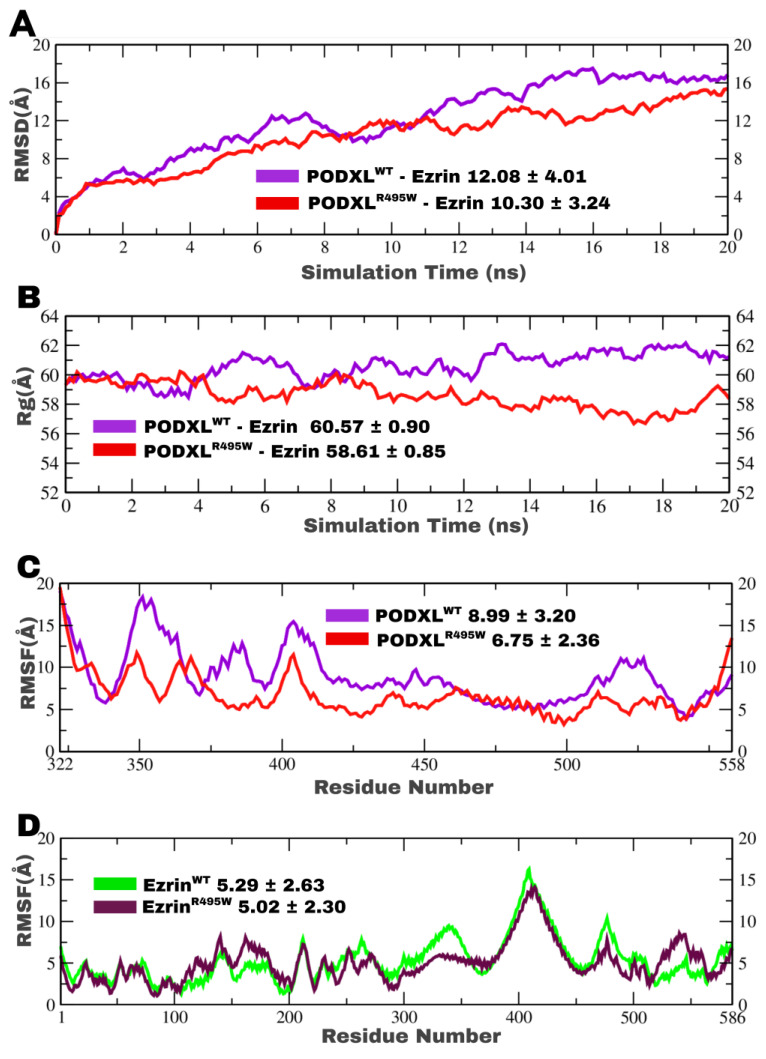
Conformational stability and flexibility analysis of PODXL^WT^–Ezrin and PODXL^R495W^–Ezrin protein complexes. (**A**) illustrates the temporal evolution of structural stability for the PODXL^WT^–Ezrin complex, depicted by a purple line with a mean value of 12.08 ± 4.01 Å, and the PODXL^R495W^–Ezrin complex, shown by a red line with a mean value of 10.30 ± 3.24 Å, over a 20 ns simulation, highlighting enhanced stability in the mutant form. (**B**) showcases the spatial compactness dynamics, with the purple line representing the PODXL^WT^–Ezrin complex at a mean value of 60.57 ± 0.90 Å and the red line denoting the mutant complex at a mean value of 58.61 ± 0.85 Å, indicating a more extended yet rigid structure induced by the mutation. (**C**) delineates residue-specific flexibility profiles across residues 322–558 for PODXL alone, with the purple line for the PODXL^WT^ at a mean value of 8.99 ± 3.20 Å and the purple line for the PODXL^R495W^ at a mean value of 6.75 ± 2.36 Å, revealing reduced local deviations around the mutation site. (**D**) presents residue-level flexibility patterns over residues 0–586, with the green line for Ezrin^WT^ at a mean value of 5.29 ± 2.63 Å and the purple line for Ezrin^R495W^ at a mean value of 5.02 ± 2.30 Å, suggesting selective dampening of fluctuations that underscore mutation-induced stabilisation at the protein–protein interface.

To understand the impact of the clinically relevant R495W mutation on the PODXL–Ezrin interaction, we conducted extensive molecular dynamics (MD) simulations of both PODXL^WT^–Ezrin and PODXL^R495W^–Ezrin. We analysed key metrics of structural stability and flexibility, including root mean square deviation (RMSD), radius of gyration (Rg), and root mean square fluctuation (RMSF). The simulations, spanning 20 ns for temporal analyses and encompassing residue-specific evaluations up to position 558 (corresponding to residues 322 to 558 in the PODXL structure), reveal mutation-induced enhancements in complex stability that align with oncogenic potentiation. The RMSD trajectories over simulation time demonstrate that the PODXL^WT^–Ezrin complex (purple) exhibits a higher average RMSD of 12.08 ± 4.01 Å, with fluctuations persisting throughout the 20 ns time frame (Figure 4A). This is indicative of greater conformational drift and less equilibrated behaviour, where the F3 subdomain remains interacting with the C-terminal domain. In contrast, the PODXL^R495W^–Ezrin complex (red) displays a reduced average RMSD of 10.30 ± 3.24 Å, converging to a stable plateau after approximately 5 ns, suggesting accelerated equilibration and enhanced overall structural integrity (Figure 4A). This deviation underscores the mutation’s role in mitigating entropic penalties at the protein–protein interface, likely through the introduction of a bulkier, hydrophobic tryptophan residue that fosters stronger van der Waals contacts or π-π stacking interactions with proximal Ezrin residues.

The PODXL^WT^–Ezrin complex maintains a lower average Rg of 60.57 ± 0.90 Å, reflecting a more compact, globular conformation, albeit with pronounced variability that implies dynamic expansion–contraction cycles potentially driven by transient dissociation events at the binding interface (Figure 4B). Conversely, the PODXL^R495W^–Ezrin complex exhibits an elevated average Rg of 58.61 ± 0.85 Å, indicative of a more extended, elongated structure, consistent with early conformational changes that foreshadow partial opening of the F3-C-terminal interface, but with markedly reduced fluctuations, signifying a rigidified ensemble (Figure 4B). This observation is consistent with the R495W substitution inducing a conformational lock that restricts intramolecular motions, possibly by altering the electrostatic landscape of PODXL’s cytoplasmic tail and enhancing its anchorage to Ezrin’s FERM domain. This rigidification may reflect a potential mechanism by which the mutation may prime Ezrin activation by destabilising the F3-C-terminal interaction, previously strong in the dormant conformation. Further granularity is provided (Figure 4C,D), which probe residue-specific deviations with the RMSF.

A proxy for localised structural fluctuations with only PODXL model structure indicates the PODXL^WT^ (purple) with an average of 8.99 ± 3.20 Å across residues 322–558 (Figure 4C). This is characterised by episodic spikes exceeding 15 Å in regions corresponding to flexible loops. PODXL^R495W^ (red), however, averages 6.75 ± 2.36 Å, with attenuated peaks, highlighting diminished deviations particularly around the mutation site (residue 495) (Figure 4C). This reduction implies that the arginine-to-tryptophan substitution propagates allosteric stabilisation, constraining hinge-like motions that could otherwise disrupt interface integrity. Complementing this, the atomic fluctuations reveal comparable profiles between Ezrin in WT (green, 5.29 ± 2.63 Å) and the mutant complex (purple, 5.02 ± 2.30 Å) (Figure 4D). The Ezrin^R495W^ mutant shows slightly less water-exposed parts, hinting at targeted flexibility reduction without overall stiffness (Figure 4D).

### 3.4. Inter-Residue Distance Analysis Reveals Key Stabilising Interactions in the PODXL^WT^–Ezrin Complex, with Arg495:HH22–Asp31:OD1 Contributing to Initial Complex Formation

**Figure 5:**
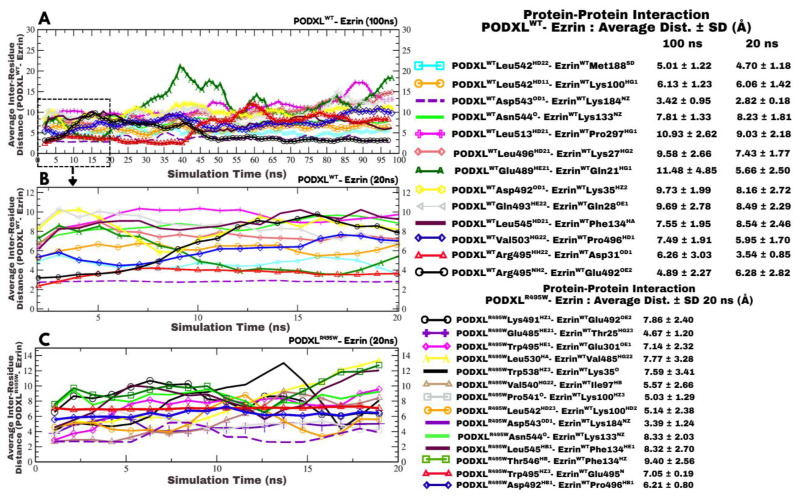
Inter-residue distance trajectories from molecular dynamics simulations of PODXL–Ezrin complexes. Plots show average interatomic distances between key residue pairs at the PODXL–Ezrin interface over simulation time (ns). The average inter-residue values (±SD) for each condition are displayed alongside the graphs. Pairs were selected for maintaining ≤ 10 Å average distances, with lines colour-coded per the right-hand legend tables (listing pairs, averages, and SD). Symbols (e.g., circles, triangles) match line styles. (**A**) PODXL^WT^–Ezrin at 100 ns, capturing long-term dynamics, especially for fluctuating pairs like Arg495^HH22^–Asp31^OD1^ (red) and Arg495^NH2^–Glu492^OE2^ (black). (**B**) PODXL^WT^–Ezrin at 20 ns, showing stable interactions (e.g., Asp543^OD1^–Lys184^NZ^, purple, mean 2.82 ± 0.18 Å) and initial dynamics. (**C**) PODXL^R495W^–Ezrin at 20 ns, highlighting mutation effects with enhanced pre-C-terminal loop contacts (e.g., Trp495^HZ3^–Glu495^N^, red, mean 7.05 ± 0.19 Å).

Molecular dynamics (MD) simulation for over 20 ns identified 22 pairs of interacting residues between the PODXL^WT^–Ezrin complex at a 3 Å distance cutoff in the initial trajectory frame. To focus on structurally and functionally relevant interactions, we refined this to 13 pairs where the average interatomic distance remained ≤10 Å over the simulation time (Figure 5). The strongest interaction was observed between PODXL^WT^Asp543^OD1^ and Ezrin^WT^Lys184^NZ^, with an average distance of 2.82 Å ± 0.18 Å over 20 ns, suggesting a robust electrostatic bond (Figure 5B). Conversely, the weakest stable pair was PODXL^WT^Leu513^HD21^ and Ezrin^WT^Pro297^HG1^, averaging ~9 Å, implying a marginal hydrophobic contact. The interatomic distance metric of interacting residue pairs revealed that three specific interactions, PODXL^WT^Asp492^OD1^–Ezrin^WT^Lys35^HZ2^, PODXL^WT^Arg495^NH2^–Ezrin^WT^Glu492^OE2^, and PODXL^WT^Gln489^HE21^–Ezrin^WT^Gln21^HG1^, exhibited dynamic behaviour (Figure 5B). Notably, the interaction between PODXL^WT^Arg495^HH22^ and Ezrin^WT^Asp31^OD1^ remained unexpectedly constant at ~4 Å over the initial 20 ns, despite the typically transient nature of such electrostatic contacts (Figure 5B). In consideration of this, we analysed the total 100 ns simulation for the PODXL^WT^–Ezrin complex, which enabled more extensive conformational sampling and probed the longevity of these dynamic interactions (Figure 5A). Specifically, we focused on those involving Arg495 and residues in Ezrin’s pre-C-terminal loop (Glu492), preceding the C-terminal domain, which is suggested to interact with the F3 subdomain, thereby stabilising the protein’s dormant structure. The further analysis of the complete simulation trajectory was justified as the initial 20 ns revealed transient fluctuations that required longer timescales to be fully characterised, ensuring robust insights into interaction stability without unnecessary computational expenditure on stable systems.

The dynamic nature of PODXL^WT^Arg495^HH22^–Ezrin^WT^Asp31^OD1^ was confirmed upon the longer 100 ns simulation (Figure 5A), with the distance increasing to ~12 Å around 59 ns, indicating hydrogen bond disruption. This positions the pair as a key transient contact essential for initial PODXL^WT^–Ezrin complex stabilisation. In contrast, PODXL^WT^ Arg495^NH2^–Ezrin^WT^Glu492^OE2^ strengthened over time, stabilising at ~4.89 ± 2.27 Å by 100 ns. Other dynamic pairs, such as PODXL^WT^Asp492^OD1^–Ezrin^WT^Lys35^HZ2^, showed complete disruption (distances > 10 Å), while PODXL^WT^Gln489^HE21^–Ezrin^WT^Gln21^HG1^ rapidly increased to ~22 Å, further supporting transient electrostatic roles. Additional disruptions occurred in pairs like PODXL^WT^Leu513^HD21^–Ezrin^WT^Pro297^HG1^, PODXL^WT^Leu496^HD21^–Ezrin^WT^Lys27^HG2^, and PODXL^WT^Gln493^HE22^–Ezrin^WT^Gln28^OE1^. PODXL^WT^Val503^HG22^–Ezrin^WT^Pro496^HD1^ weakened from average 5.95 Å to endpoint ~10 Å, diminishing the interaction with pre-C-terminal, which plausibly may preserve original F3-C-terminal interaction that maintains Ezrin in a dormant state. These findings align with our understanding that stable interactions in the PODXL^WT^–Ezrin complex maintain Ezrin’s dormant state, where F3 subdomain and C-terminal domain associations prevent premature activation, whereas transient contacts allow for regulated conformational flexibility observed during longer simulations.

### 3.5. The R495W Mutation Introduces Conformational Tension in the PODXL^R495W^–Ezrin Complex and Exposes Sites for Potential Ezrin Activation

The variable interactions of PODXL Arg495 with Ezrin residues (e.g., PODXL^WT^Arg495^HH22^–Ezrin^WT^Asp31^OD1^ and PODXL^WT^Arg495^NH2^–Ezrin^WT^Glu492^OE2^), combined with clinical evidence of the R495W missense mutation, highlighted Arg495 as a prime target for assessing mutation effects on complex stability. This substitution replaced the hydrophilic Arg495 with hydrophobic tryptophan, whose indole ring engages aromatic residues and forms weaker hydrogen bonds with polar groups, potentially triggering a conformational shift. Structural comparison revealed subtle deviations in the mutant complex, including a slightly elevated central α-helix in Ezrin and alterations in helices H8 (F2 subdomain), H11 (F3 subdomain), and H16/H18 (C-terminal region), likely due to allosteric propagation from the mutation site (Figure 3D). An atomistic indicator of mutation-induced changes was the mentioned increase in distance between Ezrin’s Pro236^HD1^ (F3 subdomain) and Thr567^HG21^ (C-terminal) (Figure S2).

### 3.6. R495W Mutation Promotes Stronger Engagement with Ezrin’s Pre-C-Terminal Loop in the PODXL^R495W^–Ezrin Complex

Analysis of the PODXL^R495W^–Ezrin complex over 20 ns revealed 14 residue pairs maintaining average distances ≤ 10 Å initially (Figure 5C). Interacting residues in Ezrin included those from the F1 subdomain (Thr25, Lys35), F2 subdomain (Ile97, Lys100, Lys133, Phe134, Lys184), post-F3 region (Glu301), and pre-C-terminal loop (Val485, Glu492, Glu495, Pro496). Compared to WT (Figure 5A,B), the mutant showed additional contacts with Ezrin Glu495 and Val485, emphasising increased engagement with the pre-C-terminal loop, located before one region (C-terminal) which is involved in the maintenance of Ezrin’s dormant state. Therefore, additional and more stable contacts at this site may render the opening of Ezrin’s closed conformation and plausibly prime it for potential activation.

The most stable pairs involved pre-C-terminal loop residues, PODXL^R495W^Trp495^HZ3^–Ezrin^WT^Glu495^N^ (~7.05 Å ± 0.19 Å average) and PODXL^R495W^ Asp492^HB1^–Ezrin^WT^Pro496^HB1^ (~6.21 Å ± 0.80 Å), highlighting mutation-enhanced stability in interactions within this region (Figure 5C). Considering its location preceding C-terminal critical for Ezrin dormant conformation, any novel interactions may lead to the disruption of F3/C-terminal interaction, thereby potentially enhancing the active state of Ezrin, which is more prone to oncogenic signalling. The fluctuating PODXL^R495W^Lys491^HZ1^–Ezrin^WT^Glu492^OE2^ stabilised at ~6 Å. However, PODXL^R495W^Leu530^HA^–Ezrin^WT^Val485^HG2^ disrupted completely (up to 14 Å). Pairs conserved from WT, such as PODXL^R495W^Asp543^OD1^–Ezrin^WT^Lys184^NZ^ (3–5 Å) and PODXL^R495W^Asn544^O^–Ezrin^WT^Lys133^NZ^ (8–10 Å), retained similar stability. Trp495 also formed a transient interaction with Ezrin’s Glu301^OE1^ (post-F3), but its stronger contact was with the pre-C-terminal loop (Glu495).

### 3.7. Virtual Screening and Hit Identification Against the Wild-Type PODXL–Ezrin Complex

To identify potential inhibitors of the protein–protein interaction between PODXL and Ezrin, a structure-based virtual screening campaign was conducted against the wild-type (WT) PODXL–Ezrin complex. A diverse chemical library of several thousand compounds was docked into the key binding interface, and the resulting poses were scored and ranked based on their predicted binding affinity and complementarity to the target site.

From this screening, six compounds emerged as top-ranking hits based on a combination of high docking scores, favourable interaction profiles (including key hydrogen bonds and hydrophobic contacts), and structural novelty. These selected hits were lapatinib, chrysin, cannabidiol, tetrahydrocannabinol (THC), NSC305787, and NSC668394. Their common names, IUPAC names, molecular weights, and canonical SMILES strings are summarised in Table 2.

Although the six ligands (Table 2) are both natural and synthetic compounds with different chemical structures, they share some important common features that may be enhancing their interaction with the PODXL–Ezrin complex. Lapatinib (PubChem CID: 208908), a man-made drug designed to block certain cancer-related proteins, is observed with complex ring structures with chlorine and fluorine atoms, which may help it stick firmly to its target. Chrysin (PubChem CID: 5281607) is a natural plant compound with a flat ring-shaped structure and groups that can form hydrogen bonds, allowing it to attach to proteins in specific ways. Cannabidiol (CBD) (PubChem CID: 644019) and tetrahydrocannabinol (THC) (PubChem CID: 16078) are both from cannabis plants and have similar structures: both have rings and long side chains with parts that can interact with proteins through hydrogen bonds and hydrophobic (water-repelling) interactions. However, they exhibit critical structural differences: THC contains a closed tricyclic ring system with a pyran (cyclic ether) ring that confers conformational rigidity. On the other hand, CBD possesses an open bicyclic structure with two free hydroxyl groups on the aromatic ring, providing greater rotational flexibility and enhanced hydrogen bonding capacity.

The other two compounds, NSC305787 (PubChem CID: 470998) and NSC668394 (PubChem CID: 381594), are synthetic molecules with fused ring systems known as quinolines. These rings, along with attached chlorine or bromine atoms and groups capable of hydrogen bonding, make these ligands effective at fitting into protein binding sites and disrupting interactions. These compounds share common chemical traits which include the flat aromatic rings that interact with proteins, groups capable of forming hydrogen bonds for site-specific attachment, and often halogen atoms like chlorine, fluorine, or bromine that enhance binding stability. Their overall size and shape are optimised to stay small enough for target access while still forming strong, stable interactions.

### 3.8. Docking Reveals Differential Binding Efficacies Against Wild-Type and Mutant PODXL–Ezrin Complexes

Virtual screening quantitatively evaluated the binding affinity of the six candidate compounds against both the PODXL^WT^–Ezrin complex and its clinically relevant mutant counterpart, PODXL^R495W^–Ezrin. The calculated binding energies (ΔG, kcal/mol) for each complex are summarised in Table 3 and reveal a compelling narrative of ligand–target interaction.

Lapatinib exhibits the strongest predicted binding affinity to both the wild-type (−8.538 kcal/mol) and, notably, the R495W mutant complex (−8.744 kcal/mol). This suggests that lapatinib possesses a robust binding mode that is not only maintained but slightly enhanced in the mutated interface. Interestingly, the docking results categorised the candidate drugs into two distinct groups based on their preference for the mutant or wild-type interface. Three compounds, lapatinib, chrysin, and NSC305787, were predicted to bind more strongly to the mutant R495W complex, as indicated by their more negative binding energies and positive percentage difference. Among these, NSC305787 is particularly noteworthy. Despite its significantly lower molecular weight (445.4 g/mol) compared to lapatinib (581.1 g/mol), it achieved a remarkably high binding affinity to the mutant complex (−8.612 kcal/mol), demonstrating the highest (+4.93%) improvement over its already strong wild-type binding.

In contrast, the other three candidates, cannabidiol, THC, and NSC668394, displayed a clear preference for the wild-type complex, with their binding affinities weakening in the presence of the R495W mutation. This was most pronounced for cannabidiol, which suffered the largest drop in binding affinity (−11.5%), resulting in the weakest interaction with the mutant complex across all tested compounds (−6.425 kcal/mol). This clear divergence in behaviour reflects the significant impact of the R495W mutation on the binding physicochemical landscape of the protein complex interface.

These selected compounds displayed differential binding via specific pockets at F2/C-terminal and F1/F3 interfaces observed through MD simulations. Lapatinib occupies the pre-C-terminal loop/H12 helix interface in Ezrin (residues 483–486/311, 318) and PODXL cytoplasmic tail, forming hydrogen bonds with Glu311 (H12), Val485 (loop), and Gly532 (PODXL^WT^) (Figure S3(Ai,Aiii,Aiv)). In the mutant, it relocates to the F2/C-terminal interface, contacting Glu120, Met558, Leu522, Ser535, and Arg562 in Ezrin, forming hydrogen bonds with the latter three, without PODXL involvement (Figure S3(Bi,Biii,Biv)), implying mutation-induced exclusion of PODXL from stabilisation. In PODXL^WT^–Ezrin, chrysin similarly binds the cytoplasmic domain/pre-C-terminal loop interface, engaging Asp536, Ser537, Ile539 (PODXL^WT^), and residues 484–488 (Ezrin) (Figure S5(Aiii,Aiv)). In the mutant form, this site is vacated; at frame 191, chrysin shifts to the F3/C-terminal interface (Met292, Lys296 Ser504, Ser505, Glu506, Gly507, Ile508 in Ezrin; Figure S4(Biii)), leading to dissociation and reduced inhibitory efficacy due to proximity to R495W.

Cannabidiol positions at the WT F1/F3 interface (Phe55, Pro56 in F1 and Arg275, Lys278, Arg279 in F3 in PODXL^WT^-Ezrin; Figure S5(Aiii)), while in the mutant, it exposes to F3 surface residues (Lys209, Gly213, Lys253, Tyr270, Pro272, Arg273, Arg509; Figure S5(Biii,Biv)), distorting PODXL H6 and limiting contacts to four residues (Val528, Ser529, Trp538, Val540; Figure S6(Bii)). NSC668394 binds the WT F1/C-terminal interface (Lys27, Leu59, Leu61, Asp62, Lys319, Thr497 in Ezrin) and Tyr507 in PODXL (Figure S6(Aiii,Aiv)), bending H6 and enabling novel PODXL–Ezrin contacts (e.g., Glu501/Lys64; Figure S7(Ai,Aii)). In the mutant, it buries at the F1/F3 interface (Trp43, Glu87, Glu92, Leu290, Arg293, Arg294, Arg295, Thr299), preserving H6 integrity (Figure S6(Bi,Biii)). Hydrogen bond frequencies corroborate these preferences, with chrysin-WT being the top performer based on the number of interactions (114; Figure S8B) and NSC668394 having the highest maximum frequency withing mutant ligand-bound complexes (37.92%, Figure S9E).

### 3.9. Structural Dynamics Analysis of Drug-Bound PODXL–Ezrin Complexes

**Figure 6:**
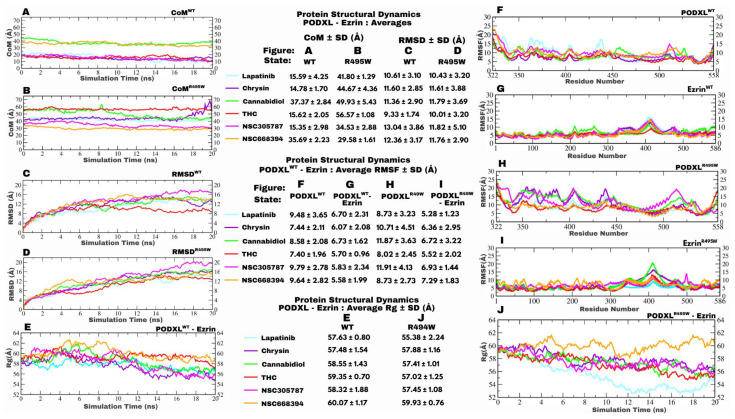
Time-dependent and residue-specific structural dynamics of PODXL–Ezrin complexes were evaluated for the wild-type (WT) and R495W mutant in the presence of lapatinib, chrysin, cannabidiol, THC, NSC305787, and NSC668394. (**A**,**B**) Centre of mass (CoM) distance trajectories for PODXL^WT^–Ezrin (**A**) and PODXL^R495W^–Ezrin (**B**). (**C**,**D**) Root mean square deviation (RMSD) profiles illustrating backbone stability for PODXL^WT^–Ezrin (**C**) and PODXL^R495W^–Ezrin (**D**). (**E**,**J**) Radius of gyration (Rg) trajectories of PODXL^WT^–Ezrin (**E**) and PODXL^R495W^–Ezrin (**J**) complexes, reflecting overall compactness. (**F**–**I**) Root mean square fluctuation (RMSF) plots showing residue-level flexibility for PODXL^WT^ (**F**), Ezrin^WT^ (**G**), PODXL^R495W^ (**H**), and Ezrin^R495W^ (**I**). Average values ± standard deviations for CoM, RMSD, RMSF, and Rg are summarised in the accompanying tables. Together, these simulations highlight the impact of the R495W mutation and small-molecule binding on the dynamic stability and conformational flexibility of PODXL–Ezrin complexes.

Structural behaviour of wild-type (WT) and R495W mutant PODXL–Ezrin complexes in both unbound and ligand-bound forms using molecular dynamics simulations was investigated. Multi-panel graphs (Figure 6) illustrate centre of mass trajectories (CoM; Figure 6A,B), root mean square deviation (RMSD; Figure 6C,D), root mean square fluctuation (RMSF; Figure 6F–I), and radius of gyration (Rg; Figure 6E,J), emphasising ligand-specific changes in stability, flexibility, and compactness that guide therapeutic strategies targeting the oncogenic interface.

### 3.10. Centre of Mass Distance Analysis Reveals Distinct Drug Positioning Between Wild-Type and Mutant Complexes

**Figure 7:**
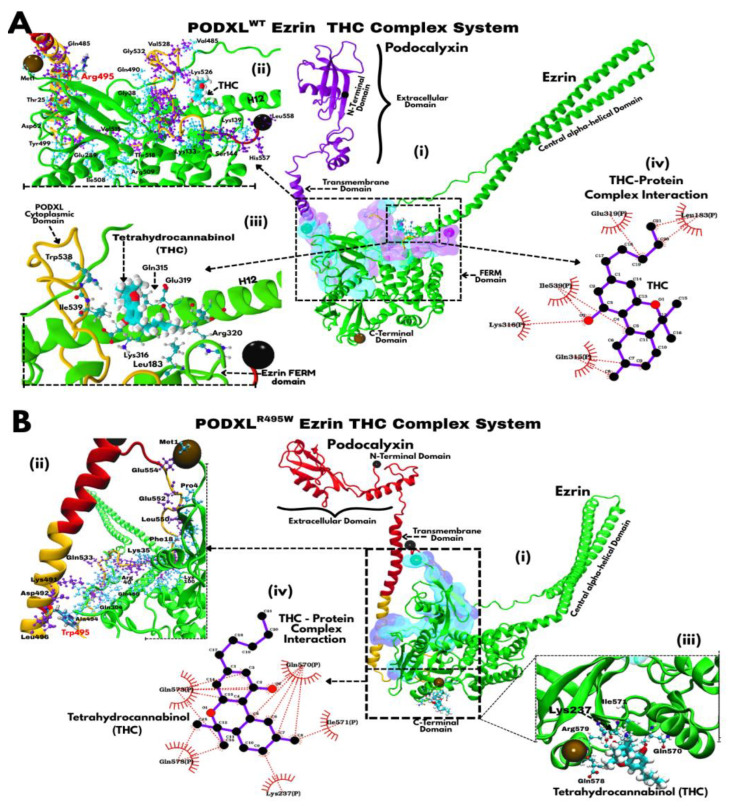
Structural representation of Tetrahydrocannabinol (THC) binding at the PODXL–Ezrin interface in PODXL–Ezrin^WT^ complex and PODXL–Ezrin^R495W^ mutant complex. (**A**) Wild-type (WT) PODXL–Ezrin–THC complex. (**i**) Overall structure showing Ezrin (green), Podocalyxin (purple), and THC (cyan balls and stick) positioned at the cytoplasmic–FERM domain interface. (**ii**) Intermolecular contacts between Podocalyxin and Ezrin depicting the core binding interface. (**iii**) THC occupying the interfacial pocket, engaging Ezrin residues Leu183, Gln315, Lys316, Glu319, Arg320, and Podocalyxin residue Trp538. (**iv**) Interaction map highlighting hydrogen bonding contacts of THC with Leu183, Gln315, Lys316, Glu319, and Ile539, stabilising the WT complex. (**B**) R495W mutant PODXL–Ezrin–THC complex. (**i**) Global architecture showing altered positioning of THC (cyan) relative to Ezrin (green) and mutant Podocalyxin (red). (**ii**) Interaction interface illustrating mutation-induced rearrangement between the two proteins. (**iii**) THC repositioned toward Ezrin’s C-terminal region. (**iv**) Interaction map showing new hydrogen bonding partners Lys273, Gln570, Ile571, Gln573, and Gln578, reflecting adaptive binding in the mutant state.

**Figure 8:**
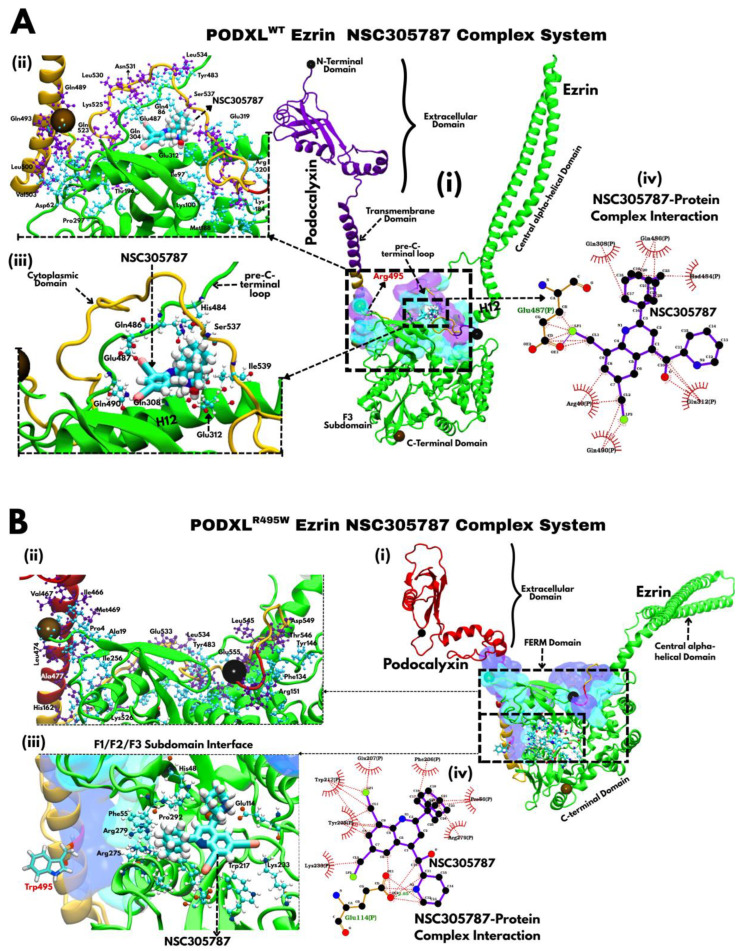
Structural representation of NSC305787 binding at the PODXL–Ezrin interface in wild-type and PODXL–Ezrin^R495W^ mutant complex. (**A**) PODXL–Ezrin^WT^–NSC305787 complex. (**i**) Overall structural orientation showing Ezrin (green), Podocalyxin (purple), and NSC305787 (cyan balls and stick) bound at the cytoplasmic–FERM domain interface. (**ii**) Detailed view of the Podocalyxin–Ezrin contact region highlighting direct intermolecular contacts. (**iii**) Binding pocket showing NSC305787 forming hydrogen bonds with Gln308, Glu312, His484, Gln486, Glu487, Gln490, and Glu312, stabilising helices H12–H13 and the pre-C-terminal loop. (**iv**) Two-dimensional interaction map illustrating hydrogen bonding and electrostatic contacts that reinforce the WT interface. (**B**) R495W mutant PODXL–Ezrin—NSC305787 complex. (**i**) Global structure showing Ezrin (green), mutant Podocalyxin (red), and NSC305787 (cyan) repositioned at the F1/F2/F3 subdomain interface. (**ii**) Residue-level view of Podocalyxin–Ezrin rearrangement induced by mutation. (**iii**) Binding pocket of NSC305787 forming hydrogen bonds with Pro56, Glu114, Tyr205, Phe206, Glu207, Tyr217, Lys233, Lys239, and Arg279, reflecting a shift toward Ezrin-directed binding. (**iv**) Interaction schematic summarising hydrogen bonding and van der Waals contacts stabilising the mutant complex. Arrows indicate zoomed-out views.

Analysis of the centre of mass (CoM) distances between the PODXL and Ezrin protein complexes and bound ligands over 20 ns molecular dynamics simulations revealed distinct binding modes and dynamic behaviours modulated by the R495W mutation (Figure 6A,B). In the PODXL^WT^–Ezrin complex, ligands engaging dual interfaces with PODXL and Ezrin, lapatinib, chrysin, THC, and NSC305787 exhibited the lowest CoM distances (Figure 6A), ranging from 8.07 to 23.1 Å (lapatinib), reflecting their proximity to the core assembly centred around PODXL’s cytoplasmic domain and Ezrin’s central α-helical domain (H12) or its boundary with the C-terminal domain (Figure 7A, Figure 8A, Figures S3A and S4A). This positioning aligns with the scaffold-stabilising role of the PODXL–Ezrin interaction in maintaining apical–basal polarity, where such ligands may reinforce the native conformation by occupying shared binding pockets identified via docking. Notably, chrysin maintained the most constant CoM trajectory (purple trace) (Figure 6A), suggesting minimal drift and potential as a stabiliser of the WT interface, consistent with its contact at the Ezrin α-helical/C-terminal boundary (pre-C-terminal loop) (Figure S4(Aiii)).

In contrast, ligands peripheral to this core, such as cannabidiol and NSC668394, displayed elevated CoM distances, with cannabidiol decreasing modestly from 44.67 Å to 37.37 Å (Figure 6A, green trace), indicative of gradual accommodation toward the Ezrin FERM domain interface (F1–F3 subdomains, residues 55–56 and 275, 278, 279; Figure S5(Ai,Aiii,Aiv)). NSC668394 also converged after an initial peak at the 10 ns mark (Figure 6A), correlating with its transient engagement of PODXL residue Y507 and the F1/C-terminal interface, which induced extensive remodelling of the PODXL interacting region (residues 483–554; Figure S6(Ai,Aiii)). THC emerges as the only ligand contacting the FERM domain (L183 in F2 subdomain; Figure 7(Aiii,Aiv)), positioning it as a WT stabiliser despite broader excursions, underscoring the heterogeneous ligand effects on the dynamic PODXL cytoplasmic tail Ezrin FERM linkage implicated in lumen formation and polarity regulation.

The R495W mutation profoundly altered these profiles, yielding uniformly higher CoM distances across all ligands (Figure 6B), consistent with the absence of PODXL contacts owing to tryptophan-induced conformational shifts that occlude the cytoplasmic site. The predominant binding shifted to Ezrin’s FERM subdomains, C-terminal domain, or their interfaces, amplifying peripheral positioning relative to the complex CoM. NSC668394 emerged as an exception, displaying comparable mean distances in both variants (35.69 Å in WT (Figure 6A) to 29.58 Å in R495W (Figure 6B)), attributable to its conserved niche between the F1 and F3 subdomains (residues 43, 87, 92, 290, 293–295, and 299; Figure S6(Bi,Biii)), which may exploit mutation-exposed pockets without reliance on PODXL. THC showed the widest separation (~56 Å; Figure 6B), shifting the engagement to C-terminal and F3 subdomain (K237) engagements (Figure 7(Biii,Biv)), whereas cannabidiol exhibited a pronounced convergence from 49.93 Å to 37.37 Å (Figure 6B), suggesting adaptive repositioning toward F3 during simulation (Figure S5(Biii)), potentially mitigating mutation-driven enhancements in oncogenic affinity.

Chrysin’s trajectory in the mutant complex highlighted instability: a stepwise increase from 49.38 to 68.34 Å, punctuated by transient reapproaches (e.g., frame 194, dropping to 56.83 Å without rebinding; Figure 6B), culminating in complete dissociation post-frame 191 (last contact near the Trp495 site; Figure S4(Biii,Biv)). This divergence from its favourable WT docking affinity (Table 3) implies that the mutation suppresses sustained interactions, transforming a potential stabiliser into a dissociator. This additionally reveals R495W’s role in selectively supressing ligand’s ability to make lasting stabilising interactions, ultimately reducing its inhibitory potential. The identified CoM patterns reveal a mutation-sensitive binding landscape: wild-type-stabilizing poses lead to peripheral, FERM-focused binding in the R495W mutant. This highlights allosteric vulnerabilities that could disrupt pro-metastatic PODXL–Ezrin signaling during epithelial-to-mesenchymal transition (EMT).

The molecular basis of Tetrahydrocannabinol (THC) binding at the PODXL–Ezrin interface was further explored due to its unique association with the F2 subdomain (Figure 7). In the wild-type configuration PODXL^WT^–Ezrin complex, THC is positioned at the junction between the Ezrin FERM/central alpha-helical domain and the cytoplasmic tail of Podocalyxin, forming a network of stabilising interactions that reinforce interdomain contact (Figure 7A). THC predominantly forms hydrogen bonds with Ezrin residues Leu183, Gln315, Lys316, Glu319, and Arg320, alongside a key hydrophobic and π-stacking association with Trp538 of Podocalyxin (Figure 7(Aiii)), establishing a dual anchoring mode that bridges both proteins. This configuration situates THC near the cytoplasmic region previously identified as the principal Podocalyxin–Ezrin binding zone, thereby reinforcing the structural integrity of the complex. Figure 7(Aii) illustrates the direct intermolecular contacts between Podocalyxin and Ezrin at the cytoplasmic–FERM domain interface.

In the PODXL^R495W^–Ezrin mutant complex (Figure 7B), THC relocates to a more distal site by interacting with residues from Ezrin’s F3 and C-terminal domains, forming an altered hydrogen bonding network with Lys273, Gln570, Ile571, Gln573, and Gln578 (Figure 7(Bi,Biii,Biv)). These residue interactions suggest a compensatory engagement on the Ezrin side, likely resulting from conformational changes induced by the R495W substitution in the Podocalyxin tail. This relocation corresponds with the broader centre of mass (CoM) shift observed for THC in PODXL^R495W^–Ezrin mutant simulations (Figure 6B), reflecting a weakened association with Podocalyxin and increased dependence on Ezrin surface residues. Despite this displacement, the interaction pattern indicates that THC can maintain interfacial stabilisation, consistent with the lower RMSD values observed for THC-bound systems in both WT and mutant states (Figure 6C,D).

NSC305787 (Figure 8) emerged as uniquely potent in modulating the PODXL–Ezrin interaction due to its dual-binding versatility and structural complementarity to both the PODXL^WT^–Ezrin complex and PODXL^R495W^–Ezrin complex interfaces. In the wild-type setup, NSC305787 docks at the boundary between Podocalyxin’s cytoplasmic tail and Ezrin’s FERM domain (Figure 8A). Among other ligands, NSC305787 maintains strong affinity for the pre-C-terminal loop and FERM domain through a dense network of polar interactions forming an extensive array of hydrogen bonds with residues including Arg40, Gln308, Glu312, His484, Gln486, Glu487, and Gln490 (notably Gln308, His484, Gln486, and Glu487; Figure 8(Aiii,Aiv)). These bonds securely tether the compound to Ezrin’s helices H12–H13 and the preceding C-terminal loop, directly stabilising the same region where Arg495 mediates Ezrin association (Figure 8(Ai)). This positions it as a functional mimic or compensator of the native PODXL–Ezrin electrostatic network, thereby locking in the local protein conformation and strengthening the overall protein–protein contact. Such effects align with the lower root mean square deviation (RMSD) variability seen in the ligand-bound form, as detailed in Section 3.9 (Figure 6C).

In the PODXL^R495W^–Ezrin complex, by contrast, NSC305787 shifts its position toward the interface between Ezrin’s F1, F2, and F3 subdomains, a change driven by the mutation’s reshaping of the binding pocket (Figure 8(Bi)). This strategic location of the drug at the key Ezrin region may allow for allosteric regulation of crucial interactions between Podocalyxin and the pre-C-terminal loop, and hence, potential regulation of dormant/active conformation as we previously suggested. NSC305787 now establishes hydrogen bonds with a new set of residues: Pro56, Glu114, Tyr205, Phe206, Glu207, Tyr217, Lys239, and Arg279 (Figure 8(Biii,Biv)). This repositioning represents an adaptive strategy that offsets the disrupted electrostatic landscape caused by the introduction of Trp495. Notably, the interacting residues are concentrated within the grooves of the FERM domain. Such relocation mirrors the patterns observed with THC in the PODXL^R495W^–Ezrin complex (Figure 7B), underscoring how interfacial energy shifts can redirect ligand behaviour. Beyond specific atomic distances, we also analysed the overall hydrogen bond interaction network at the protein–protein interface. In the THC-bound wild-type complex, a robust network was observed, with the Leu542–Glu207 pair exhibiting the highest interaction frequency (39.51%; Figure S7A). This extensive network underpins the stabilising effect of THC on the wild-type complex. In contrast, the interaction profile in the NSC305787-bound mutant complex, while still featuring a prominent Leu542–Glu207 interaction (38.11%; Figure S7B), showed a lower total number of interactions, potentially reflecting a disruption of possible activation-priming interactions involving the pre-C-terminal loop.

### 3.11. Root Mean Square Deviation Analysis Demonstrates Mutation-Dependent Drug Effects on Complex Stability

Structural changes over 20 ns through molecular dynamics simulations provide insights into the conformational stability of ligand-bound PODXL–Ezrin complexes, revealing differential responses to the R495W mutation that complement the peripheral ligand repositioning observed in centre of mass (CoM) analyses (Figure 6A,B). In the PODXL^WT^–Ezrin complex, THC binding yielded the lowest terminal RMSD (~9–10 Å, red trace, Figure 6C), indicative of enhanced rigidity at the core interface encompassing PODXL’s cytoplasmic domain and Ezrin’s central α-helical domain, consistent with THC’s unique FERM domain contact (L183; Figure 7(Aiii,Aiv)) and proximity to the complex CoM (Figure 6A), as noted previously. This stabilisation surpassed the apo WT terminal baseline (~16 Å, Figure 4A), suggesting that THC reinforces the dormant state of Ezrin, as observed in the WT complex. Conversely, NSC305787 induced the highest endpoint RMSD (17–18 Å, pink trace, Figure 6C), exceeding the apo value (Figure 4A) and aligning with its remodelling of PODXL residues 483–554 (Figure 8), implying allosteric destabilisation that could mimic PKC-mediated disassembly during polarity shift.

Lapatinib, cannabidiol, and NSC668394 elicited intermediate RMSD values (~14 Å) in the WT context (Figure 6C), despite heterogeneous binding modes: lapatinib at the dual PODXL–Ezrin core (Figure S3(Aiii)), cannabidiol at the FERM periphery (Figure S5(Aiii)), and NSC668394 bridging F1/C-terminal interfaces (Figure 6(Aiii)), highlighting a convergence toward moderate stabilisation that may preserve Ezrin’s cytoskeletal cross-linking without fully occluding oncogenic signals. Chrysin’s profile was distinctive, with a transient RMSD dip to 10.94 Å at approximately 18 ns, followed by a rebound (Figure 6C), reflecting its constant CoM proximity (Figure 6A) and potential for dynamic fluctuations at the α-helical/C-terminal boundary (Figure S4(Aiii)), which collectively positions these ligands as tuneable modulators of the WT scaffold implicated in lumen formation.

Parallel trends emerged in the R495W mutant (Figure 6D), where THC again conferred the lowest terminal RMSD (~13 Å), acting as a stabiliser relative to the apo mutant baseline at that point (~15 Å, Figure 4A), underscoring its robustness across variants, potentially by compensating for tryptophan-induced occlusion of PODXL contacts through sustained C-terminal and F3 engagements (Figure 7(Biii)). NSC305787 drove the greatest deviation (19–20 Å, Figure 6D), amplifying destabilisation beyond the apo state (Figure 4A) and WT counterpart (Figure 6C), consistent with its multi-subdomain FERM occupancy (Figure 8(Biii)) and low CoM distance (Figure 6B), which exploits mutation-exposed pockets. This destabilising behaviour of NSC305787 is particularly promising as it diminishes Podocalyxin involvement with the pre-C-terminal domain, potentially preserving F3-C-terminal autoinhibitory contacts, which we suggest may be prone to disruption in the mutant complex. Cannabidiol, chrysin, and NSC668394 converge to similarly elevated RMSD (14–17 Å, Figure 6D), with chrysin’s pronounced increase correlating with its stepwise CoM divergence (Figure 6B) and full dissociation post-frame 191 (Figure S4), transforming a WT stabiliser into a mutant disruptor near the Trp495 site.

Notably, despite the apo R495W complex exhibiting lower intrinsic average RMSD than WT (10.30 Å vs. 12.08 Å; Figure 5A), all six ligand-bound mutant trajectories displayed consistently higher deviations than their WT equivalents (Figure 6C,D), indicating amplified conformational plasticity upon perturbation. This mutation-enhanced susceptibility, for instance, NSC305787’s 2 Å RMSD escalation in R495W (Figure 6D), suggests a broadened dynamic ensemble that heightens the vulnerability to interfacial strain, which is desirable for therapeutic targeting of pro-metastatic enhancements. Residue-specific distance analyses further mechanistically underpin these effects: in WT, THC contracted the key PODXL^WT^Asn544^O^–Ezrin^WT^Lys133^NZ^ pair to 8.15 Å (Figure S1B), from 8.89 Å in the apo state (Figure 5B), while extending PODXL^WT^Arg495^NH2^–Ezrin^WT^Glu492^OE2^ to 11.61 Å (Figure S1C) from 7.4 Å in the last frame of the apo complex (Figure 5B), averting pre-C-terminal loop reinforcement and favouring F1/F2-dominant conformations that stabilise polarity without ectopic signalling. In the R495W complex, NSC305787 uniquely fractured stabilising contacts, expanding the previously stable pairs within the pre-C-terminal loop, PODXL^R495W^Trp495^HZ3^–Ezrin^WT^Glu495^N^ to 23.42 Å (frame 132; Figure S1D) and PODXL^R495W^Arg492^HB1^–Ezrin^WT^Pro496^HB1^ to 28.22 Å (frame 142; Figure S1E), and thereby disrupting Ezrin anchoring to the mutant tail; no other ligand matched this extent. NSC305787 most effectively disrupts the pre-C-terminal loop interactions, the most stable and persistent contacts in the PODXL^R495W^–Ezrin complex, making it the most potent destabiliser of these key associations (Figure S1D,E). This may, therefore, prevent any disruptions of F3-C-terminal interaction which could be induced by the R495W mutation. These findings delineate THC as a WT-preferred stabiliser and NSC305787 as a mutant-selective destabiliser, illuminating R495W’s role in sensitising the PODXL–Ezrin axis to small-molecule intervention for curtailing epithelial-to-mesenchymal transition and metastatic dissemination.

### 3.12. Root Mean Square Fluctuation Analysis Reveals Region-Specific Flexibility Modulation by Drug Binding

Root mean square fluctuation (RMSF) profiles across PODXL and Ezrin residues in ligand-bound complexes elucidated residue-level flexibility modulations, extending the global conformational insights from RMSD and CoM analyses to reveal mutation-specific hotspots vulnerable to therapeutic perturbation (Figure 6). In the PODXL^WT^–Ezrin assembly, all six ligands uniformly reduced the overall RMSF within the PODXL regions relative to the apo baseline (Figure 6F vs. Figure 4C), fostering a more rigid conformation that aligned with the core-stabilising CoM proximities (e.g., lapatinib, chrysin, THC, and NSC305787 at 8–22.7 Å; Figure 6A) and low RMSD trajectories (e.g., THC at ~9–10 Å; Figure 6C). THC emerged as the premier rigidifier, eliciting the lowest PODXL and Ezrin RMSF values (Figure 6F,G), which corroborates its FERM-contacting mode (L183; Figure 7(Aiii,Aiv)) and contraction of the PODXL^WT^Asn544^O^–Ezrin^WT^Lys133^NZ^ pair, thereby constraining the dynamic ensemble of the juxtamembrane tail to sustain physiological polarity without metastatic drift.

In contrast, the R495W mutation reversed this response, with ligand binding increasing PODXL flexibility beyond the apo levels (Figure 6H vs. Figure 4C), highlighting the enhanced susceptibility to interfacial strain, as evidenced by the higher CoM distances (Figure 6B) and RMSD elevations (Figure 6D). NSC305787 induced the most pronounced PODXL hyperflexibility (pink trace, Figure 6H), reflecting its destabilising dominance (RMSD 19–20 Å; Figure 6D) and multi-FERM occupancy (Figure 8(Biii)), which disrupts PODXL^R495W^Trp495^HZ3^–Ezrin^WT^Glu495^N^ and PODXL^R495W^Arg492^HB1^–Ezrin^WT^Pro496^HB1^ contacts (distances 23.42 Å and 28.22 Å, respectively; Figure S1D,E). Chrysin similarly increased PODXL RMSF in the mutant (Figure 6H), attributable to its dissociation and stepwise CoM divergence (49.38–68.34 Å; Figure 6B), which exposed the Trp495 locus to unchecked motion, in contrast to its WT rigidity (Figure 6A).

Ezrin-specific dynamics further delineated the variant-dependent effects. In WT Ezrin, lapatinib uniquely increased the flexibility of residues 370–450 (central α-helical domain) (Figure 6G), exceeding apo values (Figure 4D), potentially reflecting adaptive adjustments at the H12 boundary site (Figure S3(Aiii)), whereas the remainder rigidified this segment, consistent with their core CoM anchoring (Figure 6A). In R495W Ezrin, cannabidiol and chrysin reciprocally increased 370–450 flexibility (Figure 6I), aligning with their F2 and C-terminal bindings (Figures S4(Biii) and S5(Biii)) amid elevated CoM separations (Figure 6B), whereas lapatinib, THC, and NSC305787 imposed rigidity (Figure 6I). NSC668394 broadly promoted Ezrin hyperflexibility outside this region in the mutant (Figure 6I), with profiles akin to apo (elevated RMSF in 0–100 and 200–300; Figure 4D), suggesting peripheral F1–F3 bridging that preserves intrinsic dynamics without full stabilisation (Figure S6(Biii)). NSC305787 caused a notable increase in flexibility (RMSF) around residues 492–506 in the mutant protein (Figure 6I), surpassing both the mutant without ligand (Figure 4D) and the wild-type baselines (Figure 6G). This is linked to disruptions at Glu495/Pro496 and reflects the perturbation of mutation-induced possible pre-activation interactions within the pre-C-terminal loop, potentially impairing downstream signalling. Conversely, other regions became more rigid and stabilised, particularly the F3-C-terminal interface, indicating selective destabilisation (Figure 6I). The highly mobile structural ends may disrupt actin cross-linking, essential for cell polarity and movement. Most ligands reduced overall mutant flexibility, but cannabidiol specifically increased movement in the F2 region, showing a unique disrupting effect (Figure 6I). These observations highlight how the R495W mutation broadens conformational variability and suggest NSC305787 as a promising compound to target these flexible regions and selectively inhibit PODXL–Ezrin-driven cancer progression.

### 3.13. Radius of Gyration Analysis Indicates Differential Drug-Mediated Compaction Between Wild-Type and Mutant States

Radius of gyration (Rg) profiles through 20 ns simulations quantified the structural compactness of ligand-bound PODXL–Ezrin complexes, with reductions indicating contraction toward a folded state and elevations indicating extension that could impair transmembrane signalling (Figure 6E,J). These profiles extended observations of mutation-amplified dynamics, where R495W shifted ligands to peripheral CoM positions (Figure 6B), increased RMSDs (Figure 6D), and broadened RMSF hotspots (Figure 6H,I), priming the interface for selective compaction or unravelling. In the wild-type (WT) complex, NSC668394 induced Rg decline from 62.63 Å to 58.89 Å, stabilising between 58 and 59.54 Å (Figure 6E) below the apo WT baseline (Figure 4B), fostering compactness similar to its moderate RMSD (~14 Å; Figure 6C) and subdued PODXL RMSF (Figure 6F) despite peripheral CoM excursions from Y507 and F1/C-terminal remodelling (Figure S6A). This contraction may reinforce Ezrin-mediated bundling of the PODXL tail, supporting apical lumenogenesis without ectopic activation. THC similarly drove Rg reduction (Figure 6E), aligning with its premier stabilising effects (lowest RMSD 9–10 Å; minimal RMSF; core CoM at ~15 Å with FERM L183 contact; Figure 6A,C,F,G and 7(Aiii,Aiv)), which contracted the PODLX^WT^Asn544^O^–Ezrin^WT^Lys133^NZ^ pair (Figure S1B) and constrained the ensemble for polarity maintenance. Lapatinib maintained a constrained Rg range of 56–59 Å in WT (Figure 6E), matching its dual-interface CoM proximity (~15 Å) and intermediate RMSD/RMSF (Figure 6C,F,G), suggesting balanced compaction that preserves PDZ–FERM linkage for physiological cross-linking.

The R495W mutation decoupled these responses, with NSC668394 causing Rg expansion (Figure 6J) beyond the apo mutant levels (Figure 4B), yielding elongation and showing impaired compaction amid F1–F3 bridging (Figure S6(Biii)) and a conserved low CoM (average 29.58Å, Figure 6B). This extension parallels NSC668394’s Ezrin hyperflexibility (RMSF peaks in 0–100 and 200–300; Figure 6I) and suggests that tryptophan occlusion frustrates core reinforcement, potentially exposing the interface to disassembly. However, lapatinib reversed this trend in the mutant, decreasing Rg from 59.72 to 52.59 Å (Figure 6J), a hypercompact state exceeding WT equivalents (Figure 6E) and apo baselines (Figure 4B), matching its rigidifying effect on Ezrin 370–450 (low RMSF; Figure 6I) and moderate RMSD (14–17 Å; Figure 6D) despite shifted F2/C-terminal bindings (Figure S3(Biii)). Such folding may allosterically block pro-metastatic signals by over-stabilising the PODXL tail, transforming a WT modulator into a mutant-specific one. While WT favours broad compaction, the mutant shows exploitable differences, such as NSC668394-driven extension for destabilisation and lapatinib-induced hypercompaction for entrapment, offering leverage to disrupt dorsal polarity during epithelial-to-mesenchymal transition and reduce metastatic competence.

## 4. Discussion

The interaction between PODXL, a transmembrane sialomucin glycoprotein, and Ezrin, an actin cross-linking protein of the ERM family, plays a pivotal role in driving cancer cell migration, tumorigenesis, and epithelial–mesenchymal transition (EMT) [9]. Overexpression of PODXL in various malignancies, including pancreatic, breast, and renal cancers, correlates with poor prognosis and enhanced metastatic potential, largely through its ability to scaffold the plasma membrane to the actin cytoskeleton via Ezrin [3,4,5]. During EMT, PODXL–Ezrin binding at the juxtamembrane region establishes dorsal cortical polarity, facilitating endothelial transmigration and extravasation [9]. Disrupting this complex, as shown in genetic knockdown models, abolishes these pro-invasive processes, underscoring its therapeutic promise. However, the structural basis of this interaction, particularly how the identified clinically relevant mutation R495W in PODXL’s cytoplasmic tail modulates binding affinity and downstream signalling, remains elusive. This is mainly due to the lack of high-resolution experimental full-length structure from X-ray crystallography or cryo-EM data. This knowledge gap has thus rendered the PODXL–Ezrin interface “undruggable” by previous studies as its large, dynamic surface resists conventional small-molecule targeting.

To address the existing challenges, we adopted an integrated computational workflow that combined protein–protein docking, molecular dynamics (MD) simulations, and virtual screening of our in-house curated compound libraries. The study therefore aimed to understand the structural and dynamic effects of the identified R495W mutation on PODXL–Ezrin complex formation and potential Ezrin activation. We also aimed to identify the mutation-specific small-molecule inhibitors capable of disrupting or (de)stabilising these complexes for therapeutic intervention.

In this study, we successfully modelled both the wild-type (WT) and R495W mutant variants, using AlphaFold-predicted structures, and provided the first detailed structural framework for this interaction. A key validation of our models came from stereochemical analysis, which revealed a marked improvement in the Ramachandran plot statistics for PODXL upon complex formation. The percentage of residues in the most favoured regions increased from 71.2% in the isolated apo state to 91.8% in the WT complex and 91.0% in the R495W complex (Figure 2A vs. Figure 3B,C). This significant enhancement strongly suggests that the initial lower score reflects the intrinsic flexibility and disorder of PODXL’s cytoplasmic tail rather than poor model quality, and it underscores Ezrin’s role as a stabiliser of PODXL’s conformation. This finding aligns with experimental evidence that the cytoplasmic domain of PODXL (residues 483–558) is necessary and sufficient for Ezrin binding and for mediating cellular adhesion [5,8,39]. This stabilising role aligns with experimental evidence from co-immunoprecipitation and fluorescence microscopy studies, which demonstrate direct PODXL–Ezrin binding independent of NHERF adaptors, particularly in the juxtamembrane domain [8]. Moreover, our selection of dormant Ezrin was validated by its ability to engage both PODXL variants via accessible F1 and F2 subdomains, consistent with reports that Ezrin activation is unnecessary for association with partners like Aquaporin-2 [60].

A central focal point of our study is the critical role of Arg495 in mediating dynamic PODXL–Ezrin interactions. Our simulations revealed that Arg495 engages in two distinct temporally regulated interactions with Ezrin. The interaction between PODXL^WT^Arg495^HH22^ and Ezrin^WT^Asp31^OD1^ remained constant at ~4 Å for the initial 20 ns but was disrupted after ~59 ns thereafter, indicating its role in the initial docking and stabilisation of the complex (Figure 5A). In contrast, the interaction between PODXL^WT^Arg495^NH2^ and Ezrin^WT^Glu492^OE2^ strengthened over time, stabilising at ~3.5 Å by 100 ns (Figure 5A). The involvement of Glu492 is particularly significant as it resides in Ezrin’s pre-C-terminal loop, a region adjacent to the C-terminal domain that intramolecularly interacts with the F3 subdomain to maintain Ezrin in its dormant, inactive state [42,56]. The dynamic formation of a stable bond with this region in the WT complex suggests a mechanism by which PODXL binding could allosterically influence Ezrin’s activation status.

The R495W mutation exerts significant allosteric effects, remodelling the interface with the potential to favour Ezrin priming for activation. Substituting arginine’s charged guanidinium with tryptophan’s bulky indole disrupts the original Arg495-Asp31 contact but introduces novel, stable interactions: PODXL^R495W^Trp495^HZ3^-Ezrin^WT^Glu495^N^ (~7.05 Å) and PODXL^R495W^Asp492^HB1^-Ezrin^WT^Pro496^HB1^ (~6.21 Å) within Ezrin’s pre-C-terminal loop (Figure 5C). These bonds, absent in WT, correlate with enhanced complex stability, lower average RMSD (10.30 Å vs. 12.08 Å; Figure 4A), and increased average radius of gyration (Figure 4B), suggesting a more elongated, but rigid, “locked” conformation potentially poised for conformational release by PIP2 and hence, Ezrin activation [43]. Critically, the mutation increases the Ezrin Pro236^HD1^-Thr567^HG21^ distance from 2.59 Å to 3.40 Å (Figure S2B,C), which might suggest unmasking of the phosphorylation site that could predispose Ezrin to loosening F3-C-terminal autoinhibition, as inferred from RMSD shifts in these domains (~2.12 Å). This accelerated tendency toward the disruption of autoinhibitory contacts may expedite Ezrin’s response to PIP2 and kinases, promoting actin linkage, cytoskeletal remodelling, and heightened cell motility. These subtle mutation-induced changes may underpin more aggressive cancer phenotypes, as the primed state could amplify RhoA/Rac1 and PI3K-Akt signalling, enhancing a rapid EMT and invasion [9,11,12,15]. While WT PODXL remains pathogenic as per previous reports [9], its engagement with the pre-C-terminal loop in Ezrin (PODXL^WT^Arg495^NH2^-Ezrin^WT^Glu492^OE2^; Figure 5A) is strengthened only later in the simulation. In contrast, the R495W variant establishes contacts with the pre-C-terminal domain early in the simulation and maintains them throughout. This behaviour may underlie the mutant’s accelerated ability to destabilise the autoinhibitory contacts, plausibly leading to activation, which may explain its enrichment in advanced tumours. This is like other oncogenic mutations at protein–protein interfaces, such as KRAS G12C, where steric clashes enhance GTPase signalling [61,62], highlighting how single-residue changes can allosterically tune oncogenic hubs.

Our study, therefore, proposes a model in which the clinically relevant R495W mutation induces novel contacts of PODXL with the pre-C-terminal loop in Ezrin, which may represent early changes that could precede the disruption of the F3-C-terminal interface critical for maintaining the protein’s dormant state. This highlights the mutation’s potential to stabilise an activation-prone state. Consequently, we sought to destabilise the R495W complex to counteract its activation-prone tendencies to preserve Ezrin’s dormant form, while reinforcing ligand binding in the WT complex to maintain its closed conformation. In the R495W complex, NSC305787 emerged as the optimal destabiliser, inducing the highest RMSD spike (~20 Å by 20 ns, ~3 Å above apo; Figure 6D) among tested compounds. This conformational change disrupts the stabilising PODXL^R495W^Trp495^HZ3^-Ezrin^WT^Glu495^N^ (to 23.42 Å at 132nd frame; Figure S1D) and PODXL^R495W^Asp492^HB1^-Ezrin^WT^Pro496^HB1^ (to 28.22 Å at 14 ns; Figure S1E) interactions, elevating RMSF in the pre-C-terminal loop (Figure 6I) while compacting the overall structure, including the crucial F3-C-terminal interface (lower Rg; Figure 6J). Taken together, this suggests that NSC305787 possesses the ability to selectively increase the flexibility of the pre-C-terminal domain and hence, diminish stable interactions with PODXL^R495W^, which may lead to the disruption of the F3-C-terminal interface critical for Ezrin’s inactive state. In parallel, NSC305787 induces the stability of F3-C-terminal interaction, reinforcing the dormant state which cannot bind actin, subsequently being less potent in driving EMT and invasion. Such selective flexibility in destabilising interactions, which correlate with the hypothesised activation-prone state, while reinforcing autoinhibition by interface-specific rigidification, positions NSC305787 as the most promising candidate for targeting the mutant complex.

NSC305787’s proximity to PIP2-binding residues 253, 254, 262, and 263 [43] exemplified by interacting with Glu114, Tyr205, Phe206, Trp217, Lys233, and Arg279, (Figure 8(Biii)), suggests a dual mechanism which includes a direct interface disruption plus steric hindrance of PIP2 recruitment. With a binding affinity of -8.612 kcal/mol (second to lapatinib) and molecular weight of 445.4 g/mol (Table 2 and Table 3) satisfying Lipinski’s Rule of 500 Da for oral bioavailability [63], NSC305787 outperforms alternatives. Prior studies corroborate its efficacy in osteosarcoma models, where it reduces Thr567 phosphorylation and Ezrin-mediated motility [64], positioning it as a repurposable candidate for PODXL-mutated cancers.

In contrast, THC optimally stabilises the WT complex, reducing RMSD from 16.5 Å (Figure 4A) of the last 5 ns (apo) to ~9 Å (Figure 6C), the lowest among ligands. This enhanced rigidity stems from strengthened PODXL^WT^Asn544^O^–Ezrin^WT^Lys133^NZ^ (8.89 Å to 8.15 Å; Figure 5B vs. Figure S1B) and disruption of the activation-promoting PODXL^WT^Arg495^NH2^-Ezrin^WT^Glu492^OE2^ (to 11.61 Å; Figure S1C), collectively preserving F3-C-terminal autoinhibition. THC’s binding to Ezrin’s central α-helical domain and F2 subdomain (Figure 7A) likely exerts allosteric control, potentially masking activation sites and yielding the lowest RMSF across both proteins (Figure 6F–I). Known for modulating the PI3K/Akt pathway downstream of Ezrin [65], THC’s effects align with cannabinoid reports in glioma models, where it suppresses migration via ERM dysregulation [66]. Despite a slightly lower affinity (−7.437 kcal/mol) than top binders like NSC668394 (Table 3), THC’s superior stabilisation justifies selection; NSC668394, while potent, fails to reduce RMSD as effectively in WT simulations (Figure 6C). At 314.5 g/mol (Table 2), THC also meets bioavailability criteria [63], enhancing its translational appeal. Regardless of THC’s promising stabilisation of the WT complex, significant clinical barriers exist, including psychoactive effects through CB1 receptor activation, legal/regulatory restrictions, poor oral bioavailability (~6%), and potential drug–drug interactions with cancer therapeutics [67]. Additionally, immunosuppressive concerns and the risk of cognitive impairment in vulnerable cancer patients limit its direct therapeutic application [67,68]. These findings should be viewed as proof-of-concept for the binding mechanism, motivating the development of non-psychoactive THC derivatives or structurally inspired analogues that preserve the stabilising interactions while eliminating central nervous system effects [68,69]. For clinical translation, NSC305787 represents a more viable candidate, particularly for R495W mutant contexts, given its documented safety in preclinical models and lack of psychotropic activity.

Other compounds exhibited variant preferences; for example, lapatinib, with the highest mutant affinity (−9.2 kcal/mol) (Table 3), showed modest destabilisation, likely due to kinase inhibition rather than interface targeting [70,71]; its monotherapy limitations in HER2+ breast cancer underscore combination needs [71]. Chrysin preferentially stabilised WT via DGKα-FAK interference in the FERM domain, while cannabidiol mirrored THC’s pathway effects but with weaker RMSD reduction [65,66]. The superior stabilising performance of THC compared to cannabidiol, despite their structural similarities, can be attributed to THC’s rigid tricyclic framework, which may provide better shape complementarity to the binding pocket, whereas cannabidiol’s more flexible open-ring structure results in suboptimal fitting and weaker RMSD reduction. These patterns highlight the value of our screening in revealing subtype-specific liabilities, informed by the existing literature on Ezrin modulation.

Given the insight reflected from our computational approach, missing high-resolution experimental data limit atomic precision. The 20 ns simulation window was sufficient to capture equilibrium behaviour for the mutant complex, but longer timescales might reveal additional conformational rearrangements and low-frequency dynamics. Extending the simulation time and employing enhanced sampling techniques are necessary to explore a broader conformational landscape and capture additional states that may progress toward the open and activation-prone form of Ezrin. While no NMR structures exist for full-length PODXL or Ezrin, our AlphaFold models achieve near-experimental accuracy (validated against PDB: 1NI2, RMSD = 1.21Å), with future NMR studies complementing dynamic validation. Beyond establishing a structural framework, our findings directly guide future experimental validation strategies. The identified critical residues—PODXL Arg495 and Ezrin’s pre-C-terminal loop (Glu492, Glu495, Pro496)—serve as rational targets for site-directed mutagenesis studies, where SPR or ITC can quantify how the R495W substitution alters binding kinetics. Co-immunoprecipitation assays using Flag-PODXL(WT/R495W) and HA-Ezrin constructs will test our central prediction that NSC305787 selectively destabilises the mutant complex while THC stabilises the wild-type assembly. Fluorescence polarisation with PODXL peptides (483–558) can validate the predicted binding interfaces and establish dose–response profiles for lead optimisation. Importantly, FRET biosensors monitoring the Pro236-Thr567 distance would directly confirm whether R495W primes Ezrin for activation as we propose. Together, these approaches provide a systematic roadmap for translating our computational insights into targeted therapeutic interventions against PODXL-driven malignancies.

## 5. Conclusions

Our study characterised the structural basis of the oncogenic PODXL–Ezrin interaction, revealing how the R495W mutation in PODXL remodels the binding interface to potentially stabilise a “primed-for-activation” state of Ezrin. Through engagement with Ezrin’s pre-C-terminal loop, we suggest that the mutation may allosterically disrupt the protein’s dormant conformation, providing a mechanistic explanation for its association with aggressive cancer phenotypes. Our study also identified a mutation-specific dynamic behaviour of NSC305787, which effectively destabilises the mutant complex, while THC stabilises the wild-type complex, which may suppress the pro-metastatic signalling axis. We therefore established a foundational model linking PODXL–Ezrin structural dynamics to cancer pathobiology, offering a framework for rational drug design targeting this previously “undruggable” protein–protein interaction. These findings pave the way for precision medicine approaches in cancers harbouring PODXL alterations, moving beyond broad inhibition to mutation-specific intervention.

## Figures and Tables

**Table 1:** Tools and applications utilised in the study. The table provides the name, version, and purpose for each software, ensuring reproducibility of results and use of the same functions in further studies.

Tool	Version	Purpose
HADDOCK	2.4	Protein–protein docking
CASTp	3.0	Identification/Analysis of surface binding sites
UCSF Chimera	1.18	Mutagenesis, visualisation tasks
OpenMM	8.1	Analysis toolkit and simulations
GROMACS	2024.2	Analysis toolkit and simulations
CHARMM-GUI	3.7	Input generation for MD simulation tools like OpenMM and GROMACS
PyMOL	2.5	Visualisation and analysis of molecular structures
VMD	2.0.0	Visualisation and analysis of molecular structures
MoLuDock viewer	1.0	Visualisation and analysis of molecular structures
AutoDock	4.2.6	Ligand docking
MGLTools	1.5.4	Input file preparation

**Table 2:** Molecular characteristics of six selected compounds evaluated in docking studies, including their common and IUPAC names, molecular weight (g/mol), canonical SMILES notation, and 3D chemical representations. Three-dimensional chemical representations are displayed in ball and stick model, where each ball represents an atom type as follows: grey—carbon (C), white—hydrogen (H), red—oxygen (O), blue—nitrogen (N), green—chlorine (Cl), yellow—sulphur (S), light green—fluorine (F), dark red—bromine (Br).

Common Name	IUPAC Name	Molecular Weight (g/mol)	Canonical SMILES	3D Structure
Lapatinib	N-(3-chloro-4-((3-fluorophenyl)methoxy)phenyl)-6-(5-((2-methylsulfonylethylamino)methyl)furan-2-yl)quinazolin-4-amine	581.1	CS(=O)(=O)CCNCC1=CC=C(O1)C2=CC3=C(C=C2)N=CN=C3NC4=CC(=C(C=C4)OCC5=CC(=CC=C5)F)Cl	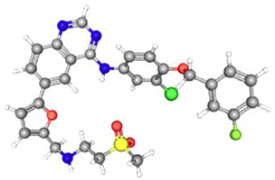
Chrysin	5,7-dihydroxy-2-phenylchromen-4-one	254.24	C1=CC=C(C=C1)C2=CC(=O)C3=C(C=C(C=C3O2)O)O	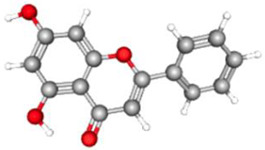
Cannabidiol	2-((1R,6R)-3-methyl-6-prop-1-en-2-ylcyclohex-2-en-1-yl)-5-pentylbenzene-1,3-diol	314.5	CCCCCC1=CC(=C(C(=C1)O)(C@@H)2C=C(CC(C@H)2C(=C)C)C)O	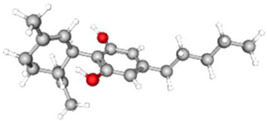
Tetrahydrocannabinol (THC)	(6aR,10aR)-6,6,9-trimethyl-3-pentyl-6a,7,8,10a-tetrahydrobenzo(c)chromen-1-ol	314.5	CCCCCC1=CC(=C2(C@@H)3C=C(CC(C@H)3C(OC2=C1)(C)C)C)O	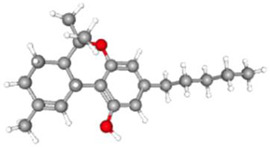
NSC305787	(2-(1-adamantyl)-6,8-dichloroquinolin-4-yl)-piperidin-2-ylmethanol	445.4	C1CCNC(C1)C(C2=CC(=NC3=C2C=C(C=C3Cl)Cl)C45CC6CC(C4)CC(C6)C5)O	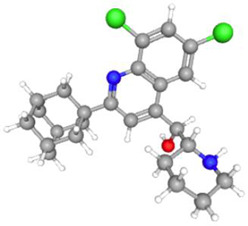
NSC668394	7-(2-(3,5-dibromo-4-hydroxyphenyl)ethylamino)quinoline-5,8-dione	452.1	C1=CC2=C(C(=O)C(=CC2=O)NCCC3=CC(=C(C(=C3)Br)O)Br)N=C1	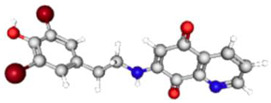

**Table 3:** Comparative binding affinities of candidate compounds for wild-type and R495W mutant PODXL–Ezrin complexes.

Candidate Drugs	Binding Affinity in PODXL^WT^–Ezrin Complex (kcal/mol)	Binding Affinity in PODXL^R495W^–Ezrin Complex (kcal/mol)	Difference in Binding Affinity Between PODXL^R495W^–Ezrin and PODXL^WT^–Ezrin (%)
Lapatinib	−8.538	−8.744	+2.41
Chrysin	−7.348	−7.638	+3.94
Cannabidiol	−7.260	−6.425	−11.50
Tetrahydrocannabinol (THC)	−7.437	−7.157	−3.76
NSC305787	−8.207	−8.612	+4.93
NSC668394	−7.775	−7.101	−8.67

## Data Availability

The original contributions presented in this study are included in the article/Supplementary Material. Further inquiries can be directed to the corresponding author.

## Supplementary Materials

The following supporting information can be downloaded at: https://www.mdpi.com/article/10.3390/jox16010025/s1. Figure S1: (**A**) CASTp-predicted surface binding sites in PODXL^WT^-Ezrin complex. Active residues selected in PODXL^WT^ (purple) are amino acids 483–554. Based on CASTp prediction, 40 residues was selected in Ezrin, out of which 6 is located in F1 subdomain: Met12, Asp13, Thr36, Ile37, Gly38, Arg40 (orange) and 34 in F2 subdomain: Ala90, Glu91, Glu92, Leu93, Ile94, Gln95, Asp96, Ile97, Gln99, Lys100, Phe103, Leu104, Lys107, Ile110, Leu111, Ala122, Leu125, Gly126, Ala129, Val130, Ala132, Lys133, Glu150, Arg151, Leu152, Leu153, Pro154, Leu183, Lys184, Asp185, Asn186, Met188, Leu189, Tyr191 (coloured in blue). (**B**) Distance between PODXL^WT^Asn544^O^ and Ezrin^WT^Lys133^NZ^ in the last frame of simulation of THC-bound wild-type complex measuring 8.15 Å. (**C**) Shows a 11.61 Å distance between residue pair PODXL^WT^Arg495^NH2^-Ezrin^WT^Glu492^OE2^ in the last frame of 20 ns simulation of WT complex bound to THC. (**D**) depicts a novel distance (23.42 Å) in 132nd frame of simulation between PODXL^WT^Trp495^HZ3^ and Ezrin^WT^Glu495^N^ pair induced by the binding of NSC305787. (**E**) NSC305787-induced change in distance in PODXL^WT^Arg492^HB1^-Ezrin^WT^Pro496^HB1^ at frame 142 is shown, equaling 28.22 Å. Figure S2: Structural analysis of F3-C-terminal interaction in PODXL^WT^-Ezrin and PODXL^R495W^-Ezrin protein complexes. (**A**) depicts the pairwise structural alignment of F3/C-terminal domain between WT and mutant complex, demonstrating an RMSD of 2.12 Å. Both F3 and C-terminal domains are colour coded between two model complexes (legend provided), facilitating straightforward comparison. (**B**) shows a distance of 2.59 Å between Pro236^HD1^ and Thr567^HG21^ in the last frame of the 20 ns simulation. (**C**) in mutant complex the Pro236^HD1^-Thr567^HG21^ distance is slightly higher, measuring 3.40 Å in the last frame of 20 ns simulation. Figure S3: Structure of lapatinib-bound PODXL^WT^-Ezrin and PODXL^R495W^-Ezrin protein complexes, highlighting drug-binding pockets and interacting residues. (**A**) Wild-type complex bound to lapatinib. (**i**) Overall structure showing lapatinib binding to PODXL^WT^-Ezrin complex. (**ii**) Interactions between residues in Podocalyxin (purple) and Ezrin (green) depict the protein complex binding interface. (**iii**) Lapatinib is located at the interface of pre-C-terminal loop and H12 helix of Ezrin and cytoplasmic domain of PODXL encompassing residues ≈ 526–539 (**iv**) It is observed that lapatinib interacts via hydrogen bonds with Glu311 (found at H12), Val485 (pre-C-terminal loop), and Gly532 (PODXL cytoplasmic domain). (**B**) PODXL^R495W^-Ezrin complex bound to lapatinib. (**i**) Global structure of ligand-bound complex shows positioning of lapatinib (cyan) relative to mutant Podocalyxin (red) and Ezrin (green). (**ii**) View of the interaction interface between Podocalyxin and Ezrin. (**iii**) Lapatinib is positioned at the F2 subdomain/C-terminal domain interface, making contacts with Glu522, Ser535, and Arg562 in Ezrin, and no residues in PODXL. (**iv**) Interaction map illustrates the contacts between lapatinib and Ezrin. Figure S4: Structure of chrysin-bound PODXL^WT^-Ezrin and PODXL^R495W^-Ezrin protein complexes, highlighting drug-binding pockets and interacting residues. (**A**) PODXL^WT^-Ezrin in complex with chrysin. (**i**) Global structure reflecting position of chrysin (cyan) relative to Podocalyxin (purple) and Ezrin. (**ii**) Interaction interface depicts key residue contacts and position of chrysin. (**iii**) Zoomed insert shows that chrysin occupies the interface between the PODXL cytoplasmic domain and the Ezrin pre-C-terminal loop in the wild-type complex, with proximity to the protein-protein interaction interface (**ii**). (**iv**) Chrysin predominantly engages in the interaction with Ser537 (PODXL cytoplasmic domain), as well as its nearby residues Asp536 and Ile 539, while contacting residues 484–488 in Ezrin. (**B**) Chrysin-bound mutant complex. (**i**) Overall complex structure with Podocalyxin in red, Ezrin in green, and chrysin in cyan. (**ii**) Cytoplasmic domain/pre-C-terminal loop interface remains unoccupied. (**iii**) At frame 191 chrysin is found at the interface of F3 subdomain and C-terminal domain in Ezrin, interacting with Met292 (in F3), and C-terminal residues Glu506 and Ile508. These are the last residues chrysin engages with in PODXL^R495W^-Ezrin protein complex, before completely disassociating, implying that R495W mutation, found near its binding pocket, suppresses chrysin’s ability to make lasting stabilising interactions, ultimately reducing its inhibitory potential. (**iv**) Interaction map confirms these findings, with chrysin making hydrogen contacts with Met292, Glu506, and Ile508. Figure S5: Structure of cannabidiol-bound PODXL^WT^-Ezrin and PODXL^R495W^-Ezrin protein complexes, highlighting drug-binding pockets and interacting residues. (**A**) PODXL^WT^-Ezrin protein complex in the last frame of simulation when bound to cannabidiol. (**i**) The overall structure reflects conformational changes induced by binding of cannabidiol. (**ii**) The PODXL^WT^-Ezrin interaction interface. (iii) This drug is seen positioned in the F1/F3 subdomain interface, closely interacting with Arg275, Lys278, and Arg279 in Ezrin. (**iv**) Two-dimensional map reflects the same interactions between cannabidiol and Ezrin. (**B**) Mutant PODXL-Ezrin-cannabidiol complex. (**i**) Global structure depicts conformational changes in complex upon interaction with cannabidiol. (**ii**) Interestingly, upon cannabidiol binding in the PODXL^R495W^-Ezrin complex, the cytoplasmic domain helix (H6) becomes distorted around the region of R495W mutation, resulting in protein-protein interaction interface becoming disrupted, with only 4 Podocalyxin residues (Val528, Ser529, Trp538, Val540) interacting with Ezrin. (**iii**) Cannabidiol is repositioned to a surface-exposed region, engaging in interactions with residues Lys209, Lys253, Tyr270, Pro272, Arg273 found in the F3 subdomain. (**iv**) Interaction map confirms interactions with Lys209, Tyr270, Pro272, and Arg273. Figure S6: Structure of NSC668394-bound PODXL^WT^-Ezrin and PODXL^R495W^-Ezrin protein complexes, highlighting drug-binding pockets and interacting residues. (**A**) NSC668394 bound to the wild—type PODXL-Ezrin complex. (**i**) The overall complex structure with positioning of NSC668394 relative to protein complex. (**ii**) Helix H6 is bent around from the region of Arg495 downwards, inducing a conformational change in PODXL^WT^-Ezrin interaction interface, where some residues in Podocalyxin cytoplasmic domain, such as Glu501 and Glu504 engage in interactions with residues in F1 subdomain of Ezrin, such as Lys64. This interaction was previously uncharacterised in our analysis with other drugs. (**iii**) The binding pocket of small molecule NSC668394 is depicted as F1/C-terminal domain interface, allowing the drug to predominantly interact with Ezrin residues located in F1 (e.g., Lys27, Leu59, Leu61, Asp62) and C-terminal (Thr497) domains, while engaging with only one amino acid in PODXL^WT^ cytoplasmic domain, Tyr507. (**iv**) The interaction map presents the contacts between NSC668394 and residues in complex. (**B**) Mutant PODXL-Ezrin-NSC668394 complex. (**i**) Overall structure depicts the position of NSC668394 (cyan) relative to mutant Podocalyxin (red) and Ezrin (green). (**ii**) The protein-protein interaction interface in mutant complex closely resembles the one of majority of drugs, where helix H6 is not distorted. (**iii**) In contrast to surface-exposed binding site in WT model complex, in PODXL^R495W^-Ezrin, NSC668394 shifts its position to a more buried site, located at the F1/F3 subdomain interface, plausibly due to potential mutation-induced conformational changes. (**iv**) The residues that contact NSC668394 include Trp43, Glu87, Glu92 (F1) and Leu290, Arg293, Arg294, Thr299 (F3), as per the interaction map. Figure S7: Inter-residue contact frequencies between PODXL and Ezrin. (**A**) Bar plot shows the frequencies for multiple residue-residue interactions in PODXL^WT^-Ezrin complex bound to THC. Interaction between Leu542 and Glu207 has the highest occurrence, being present in 39.51% of the simulation frames. (**B**) The graph shows the same residue pair, Leu542 and Glu207, to be interacting most frequently (38.11%) in NSC305787-bound PODXL^R495W^-Ezrin protein complex. Additional statistics (total interaction, maximum frequency, minimum frequency, and average frequency) are displayed for both complexes. Figure S8: Bar plots showing most frequent hydrogen bond interactions between residues in PODXL^WT^-Ezrin protein complex upon binding of (**A**) lapatinib, (**B**) chrysin, (**C**) cannabidiol, (**D**) NSC305787, and (**E**) NSC668394. Values for total number of interactions, maximum, minimum, and average frequency are displayed for each complex. (**B**) Chrysin-bound WT complex is the one with the highest number of interactions (114). (**E**) The NSC668394-bound complex exhibits the highest maximum frequency (36.91%). Figure S9: Bar plots showing most frequent hydrogen bond interactions between residues in PODXL^R495W^-Ezrin protein complex upon binding of (**A**) lapatinib, (**B**) chrysin, (**C**) cannabidiol, (**D**) THC, and (**E**) NSC668394. Values for total number of interactions, maximum, minimum, and average frequency are displayed for each complex. (**B**) The lapatinib-bound WT complex is the one with the highest number of interactions (95). (**E**) The NSC668394-bound complex exhibits the highest maximum frequency (37.92%).

## Abbreviations

The following abbreviations are used in this manuscript:

PODXL	Podocalyxin
WT	Wild-type
R495W/MT	Substitution of Arg495 to Trp495 in PODXL/mutation/sometimes referred to as the whole mutant complex
PODXL^WT^–Ezrin	Wild-type complex
PODXL^R495W^–Ezrin	Mutant complex
PODXL^MT^–Ezrin	Mutant complex
MD Simulations	Molecular dynamics simulations
RMSD	Root mean square deviation
RMSF	Root mean square fluctuation
Rg	Radius of gyration
CoM	Centre of mass
THC	Tetrahydrocannabinol
