# Supplementary material for: Structural and Dynamic Insights into Podocalyxin–Ezrin Interaction as a Target in Cancer Progression
Source: J Xenobiot. 2026 Feb 2;16(1):25. doi: 10.3390/jox16010025 (PMC12922080; doi:10.3390/jox16010025)
Supplement: Supplementary file 1 [file jox-16-00025-s001.zip › jox-4043440-supplementary.pdf]

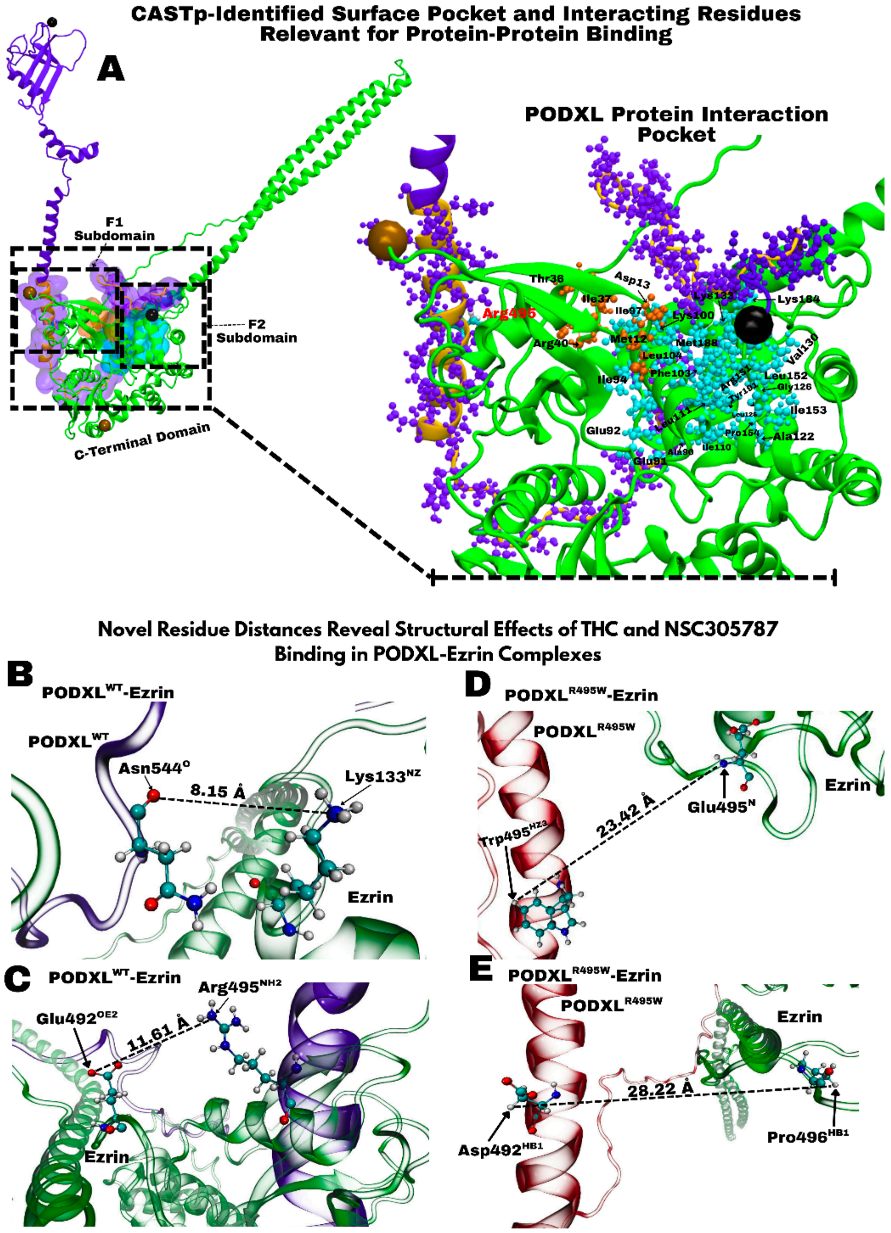

Figure S1. **(A)** CASTp-predicted surface binding sites in PODXL^{WT}-Ezrin complex. Active residues selected in PODXL^{WT} (purple) are amino acids 483-554. Based on CASTp prediction, 40 residues was selected in Ezrin, out of which 6 is located in F1 subdomain: Met12, Asp13, Thr36, Ile37, Gly38, Arg40 (orange) and 34 in F2 subdomain: Ala90, Glu91, Glu92, Leu93, Ile94, Gln95, Asp96, Ile97, Gln99, Lys100, Phe103, Leu104, Lys107, Ile110, Leu111, Ala122, Leu125, Gly126, Ala129, Val130, Ala132, Lys133, Glu150, Arg151, Leu152, Leu153, Pro154, Leu183, Lys184, Asp185, Asn186, Met188, Leu189, Tyr191 (coloured in blue). **(B)** Distance between PODXL^{WT}Asn544^{O} and Ezrin^{WT}Lys133^{NZ} in the last frame of simulation of THC-bound wild-type complex measuring 8.15 Å. **(C)** Shows a 11.61 Å distance between residue pair PODXL^{WT}Arg495^{NH2}-Ezrin^{WT}Glu492^{OE2} in the last frame of 20 ns simulation of WT complex bound to THC. **(D)** depicts a novel

distance (23.42 Å) in 132nd frame of simulation between PODXL^{WT}Trp495^{HZ3} and Ezrin^{WT}Glu495^{N} pair induced by the binding of NSC305787. **(E),** NSC305787-induced change in distance in PODXL^{WT}Arg492^{HB1}-Ezrin^{WT}Pro496^{HB1} at frame 142 is shown, equaling 28.22 Å.

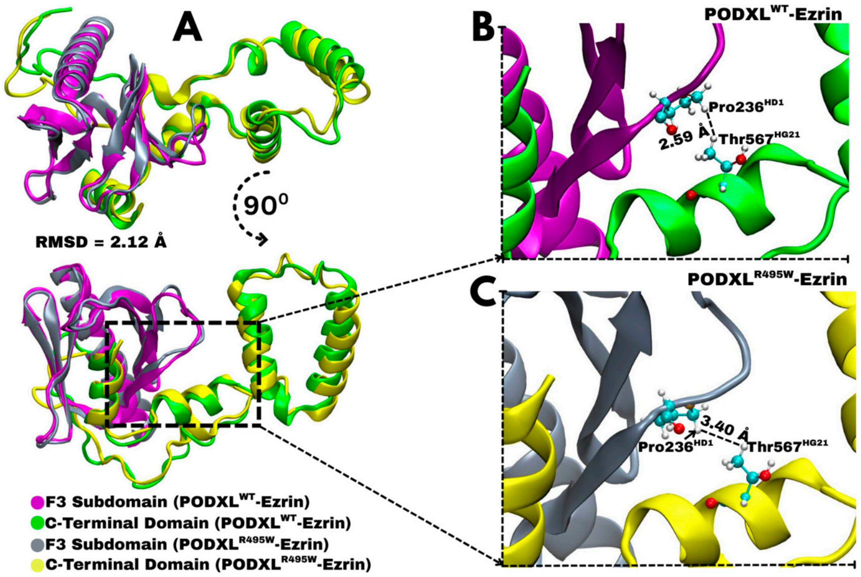

Figure S2. Structural analysis of F3-C-terminal interaction in PODXL^{WT}-Ezrin and PODXL^{R495W}-Ezrin protein complexes. **(A)** depicts the pairwise structural alignment of F3/C-terminal domain between WT and mutant complex, demonstrating an RMSD of 2.12 Å. Both F3 and C-terminal domains are colour coded between two model complexes (legend provided), facilitating straightforward comparison. **(B)** shows a distance of 2.59 Å between Pro236^{HD1} and Thr567^{HG21} in the last frame of the 20 ns simulation. **(C)** in mutant complex the Pro236^{HD1}-Thr567^{HG21} distance is slightly higher, measuring 3.40 Å in the last frame of 20 ns simulation.

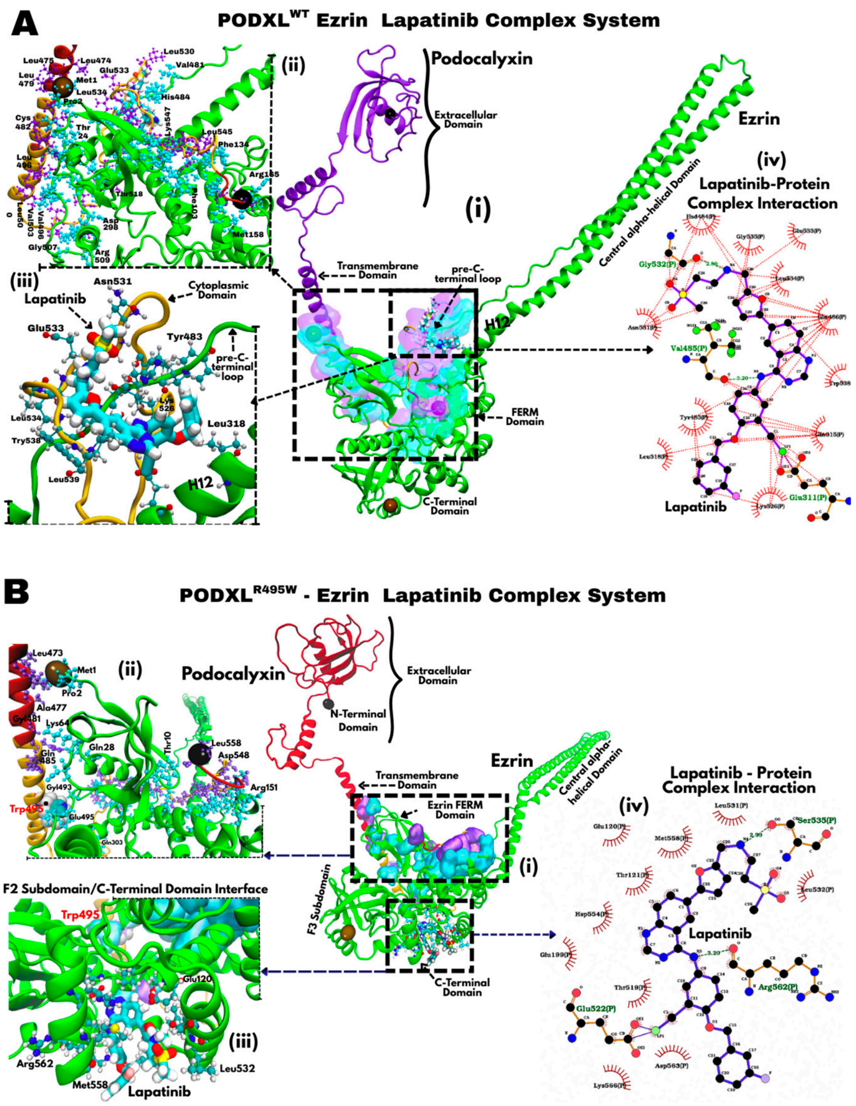

Figure S3. Structure of lapatinib-bound PODXL^{WT}-Ezrin and PODXL^{R495W}-Ezrin protein complexes, highlighting drug-binding pockets and interacting residues. (**A**) Wild-type complex bound to lapatinib. **(i)** Overall structure showing lapatinib binding to PODXL^{WT}-Ezrin complex. (ii) Interactions between residues in Podocalyxin (purple) and Ezrin (green) depict the protein complex binding interface. (iii) Lapatinib is located at the interface of pre-C-terminal loop and H12 helix of Ezrin and cytoplasmic domain of PODXL encompassing residues ≈ 526-539(iv) It is observed that lapatinib interacts via hydrogen bonds with Glu311 (found at H12), Val485 (pre-C-terminal loop), and Gly532 (PODXL cytoplasmic domain). (**B**) PODXL^{R495W}-Ezrin complex bound to lapatinib. **(i)** Global structure of ligand-bound complex shows positioning of lapatinib (cyan) relative to mutant Podocalyxin (red) and Ezrin (green). **(ii)** View of the interaction interface between Podocalyxin and Ezrin. **(iii)** Lapatinib is positioned at the F2 subdomain/C-terminal domain interface, making contacts with Glu522, Ser535, and Arg562 in Ezrin, and no residues in PODXL. (iv) Interaction map illustrates the contacts between lapatinib and Ezrin.

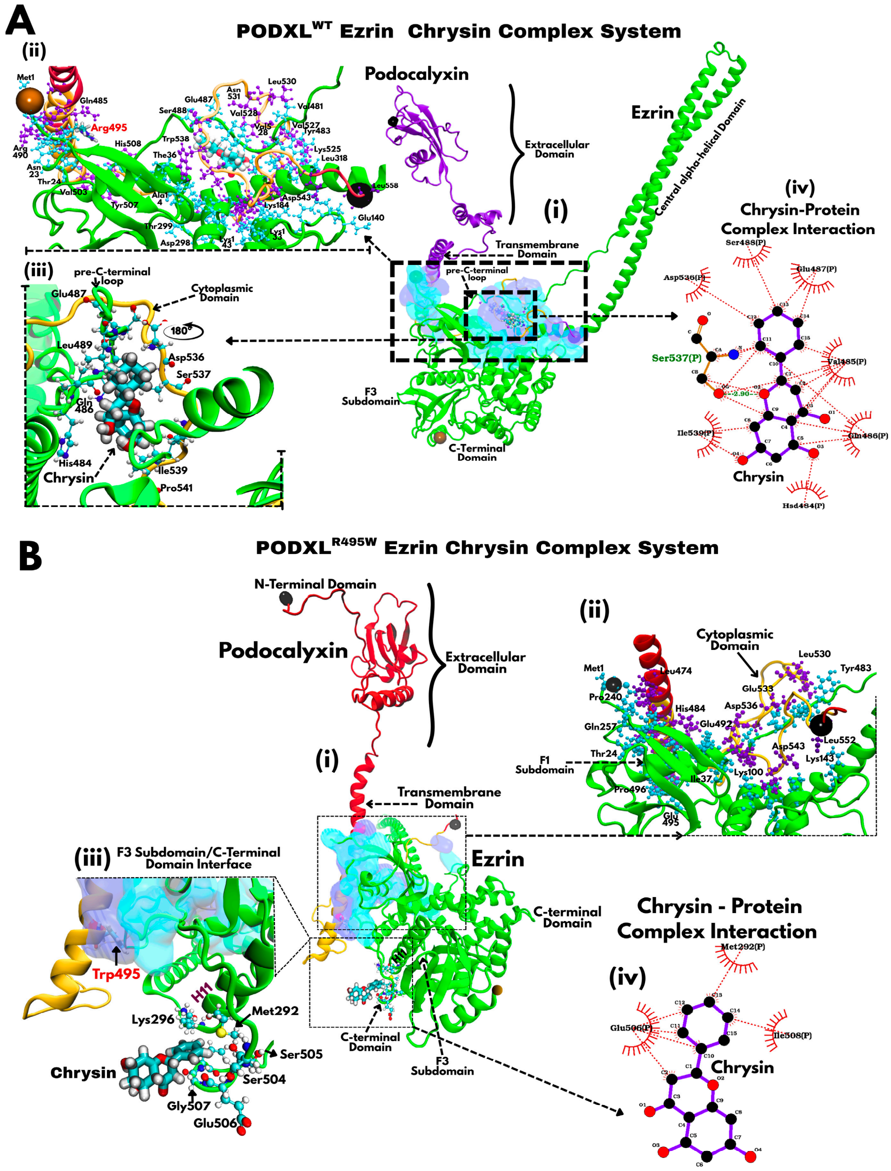

Figure S4. Structure of chrysin-bound PODXL^{WT}-Ezrin and PODXL^{R495W}-Ezrin protein complexes, highlighting drug-binding pockets and interacting residues. **(A)** PODXL^{WT}-Ezrin in complex with chrysin. **(i)** Global structure reflecting position of chrysin (cyan) relative to Podocalyxin (purple) and Ezrin. **(ii)** Interaction interface depicts key residue contacts and position of chrysin. **(iii)** Zoomed insert shows that chrysin occupies the interface between the PODXL cytoplasmic domain and the Ezrin pre-C-terminal loop in the wild-type complex,with proximity to the protein-protein interaction interface (ii). **(iv)** Chrysin predominantly engages in the interaction with Ser537 (PODXL cytoplasmic domain), as well as its nearby residues Asp536 and Ile 539, while contacting residues 484-488 in Ezrin. **(B)** Chrysin-bound mutant complex. **(i)** Overall complex structure with Podocalyxin in red, Ezrin in green, and chrysin in cyan. **(ii)**

Cytoplasmic domain/pre-C-terminal loop interface remains unoccupied **(iii)** At frame 191 chrysin is found at the interface of F3 subdomain and C-terminal domain in Ezrin, interacting with Met292 (in F3), and C-terminal residues Glu506 and Ile508. These are the last residues chrysin engages with in PODXL^{R495W}-Ezrin protein complex, before completely disassociating, implying that R495W mutation, found near its binding pocket, suppresses chrysin's ability to make lasting stabilising interactions, ultimately reducing its inhibitory potential. **(iv)** Interaction map confirms these findings, with chrysin making hydrogen contacts with Met292, Glu506, and Ile508.

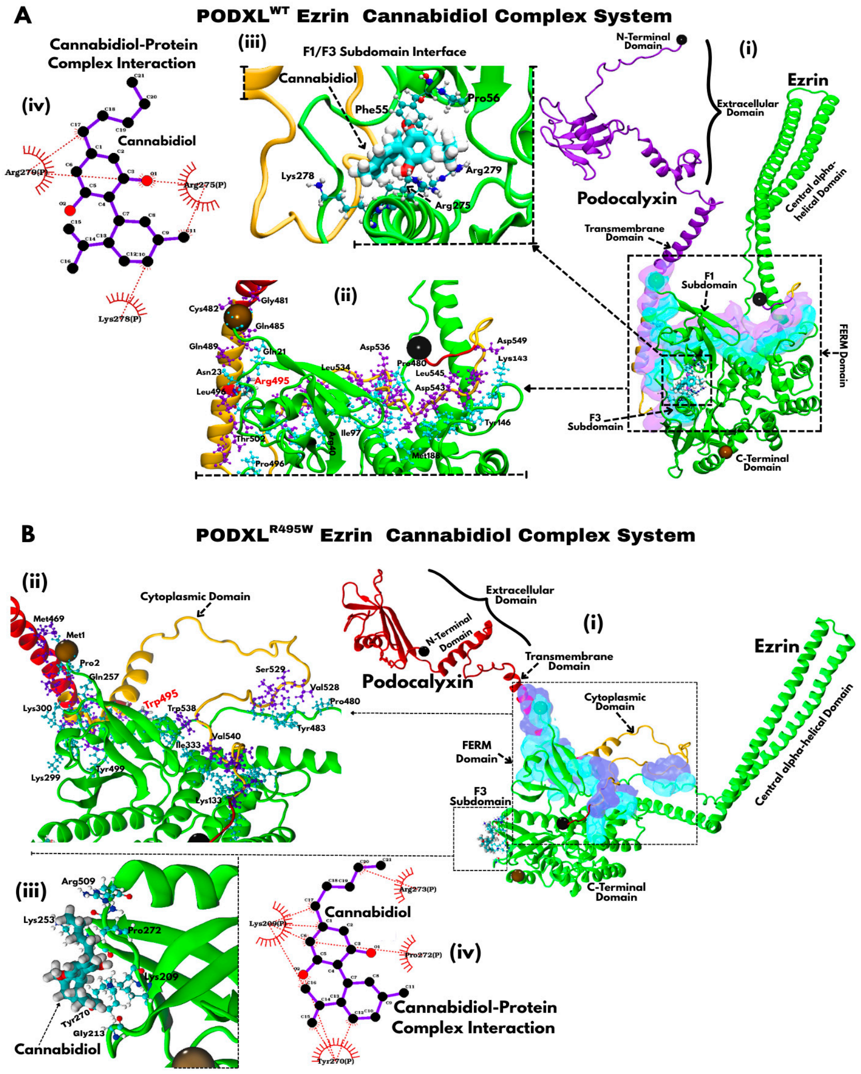

Figure S5. Structure of cannabidiol-bound PODXL^{WT}-Ezrin and PODXL^{R495W}-Ezrin protein complexes, highlighting drug-binding pockets and interacting residues. **(A)** PODXL^{WT}-Ezrin protein complex in the last frame of simulation when bound to cannabidiol. **(i)** The overall structure reflects conformational changes induced by binding of cannabidiol. **(ii)**The PODXL^{WT}-Ezrin interaction interface. (iii) This drug is seen positioned in the F1/F3 subdomain interface, closely interacting with Arg275, Lys278, and Arg279 in Ezrin. **(iv)** Two-dimensional map reflects the same interactions between cannabidiol and Ezrin. **(B)** Mutant

PODXL-Ezrin-cannabidiol complex. **(i)** Global structure depicts conformational changes in complex upon interaction with cannabidiol. **(ii)** Interestingly, upon cannabidiol binding in the PODXL^{R495W}-Ezrin complex, the cytoplasmic domain helix (H6) becomes distorted around the region of R495W mutation, resulting in protein-protein interaction interface becoming disrupted, with only 4 Podocalyxin residues (Val528, Ser529, Trp538, Val540) interacting with Ezrin. **(iii)** Cannabidiol is repositioned to a surface-exposed region, engaging in interactions with residues Lys209, Lys253, Tyr270, Pro272, Arg273 found in the F3 subdomain. **(iv)** Interaction map confirms interactions with Lys209, Tyr270, Pro272, and Arg273.

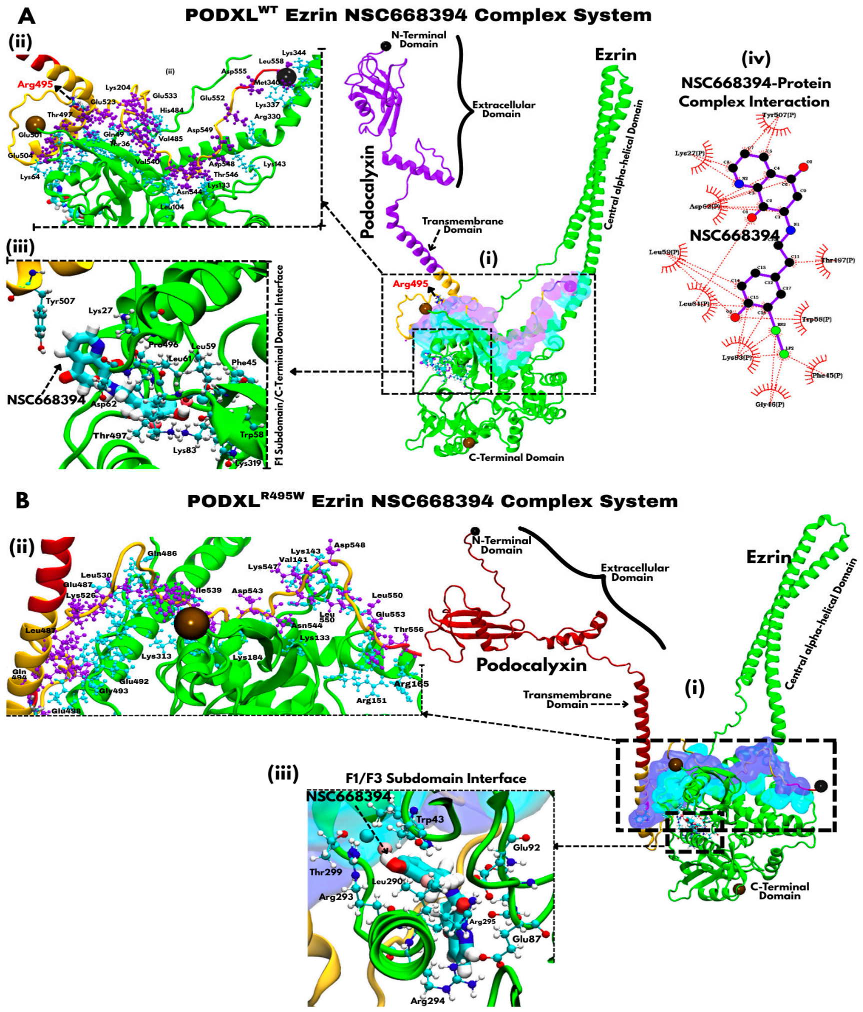

Figure S6. Structure of NSC668394-bound PODXL^{WT}-Ezrin and PODXL^{R495W}-Ezrin protein complexes, highlighting drug-binding pockets and interacting residues. **(A)** NSC668394 bound to the wild—type PODXL-Ezrin complex. **(i)** The overall complex structure with positioning of NSC668394 relative to protein complex. **(ii)** Helix H6 is bent around from the region of Arg495 downwards, inducing a conformational change in PODXL^{WT}-Ezrin interaction interface, where some residues in Podocalyxin cytoplasmic domain, such as Glu501 and Glu504 engage in interactions with residues in F1 subdomain of Ezrin, such as Lys64. This interaction was previously uncharacterised in our analysis with other drugs. **(iii)** The binding pocket of small molecule NSC668394 is depicted as F1/C-terminal domain interface, allowing

the drug to predominantly interact with Ezrin residues located in F1 (e.g. Lys27, Leu59, Leu61, Asp62) and C-terminal (Thr497) domains, while engaging with only one amino acid in $PODXL^{WT}$ cytoplasmic domain, Tyr507. **(iv)** The interaction map presents the contacts between NSC668394 and residues in complex. **(B)** Mutant PODXL-Ezrin-NSC668394 complex. **(i)** Overall structure depicts the position of NSC668394 (cyan) relative to mutant Podocalyxin (red) and Ezrin (green). **(ii)** The protein-protein interaction interface in mutant complex closely resembles the one of majority of drugs, where helix H6 is not distorted. **(iii)** In contrast to surface-exposed binding site in WT model complex, in $PODXL^{R495W}$-Ezrin, NSC668394 shifts its position to a more buried site, located at the F1/F3 subdomain interface, plausibly due to potential mutation-induced conformational changes. **(iv)** The residues that contact NSC668394 include Trp43, Glu87, Glu92 (F1) and Leu290, Arg293, Arg294, Thr299 (F3), as per the interaction map. .

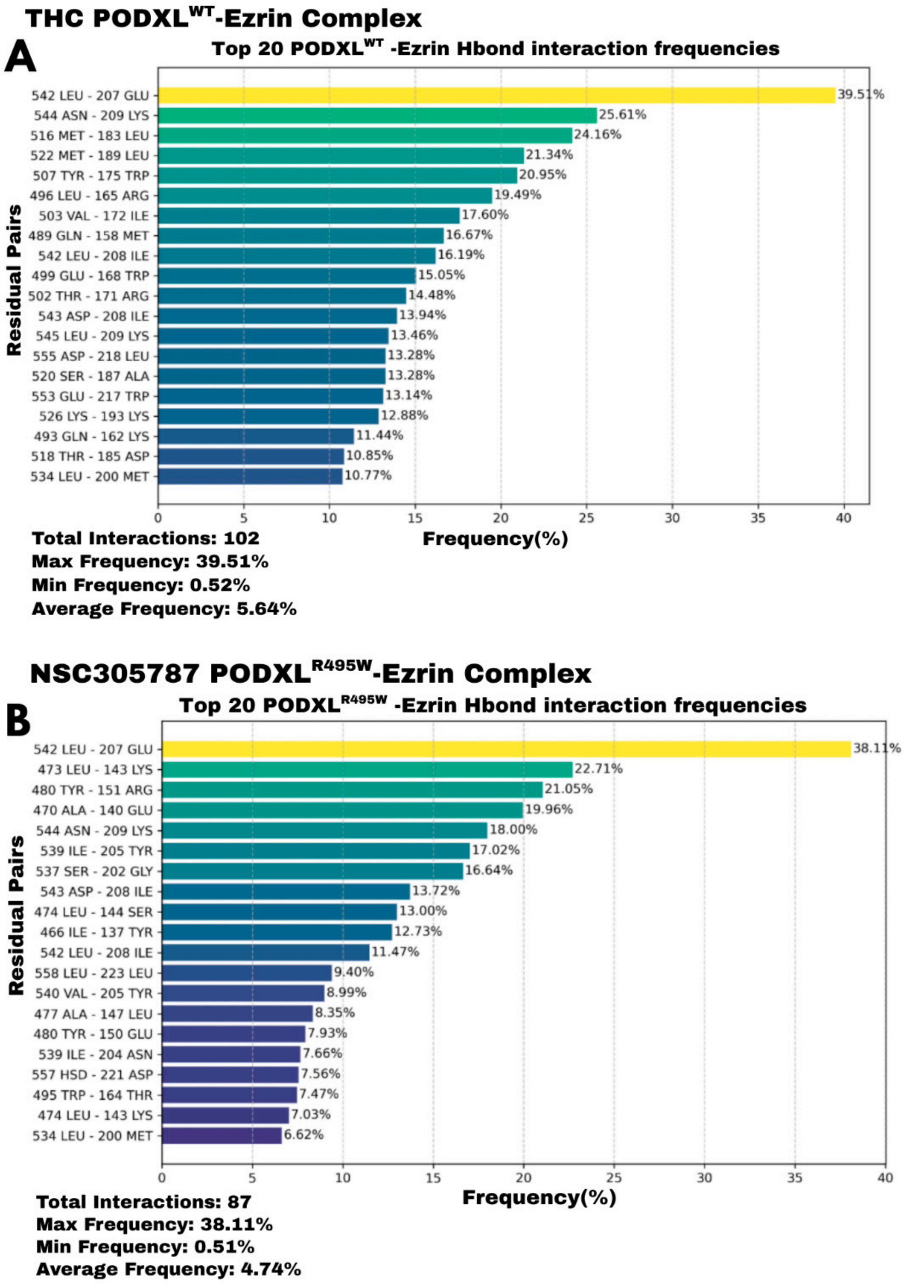

Figure S7. Inter-residue contact frequencies between PODXL and Ezrin. **(A)** Bar plot shows the frequencies for multiple residue-residue interactions in PODXL^{WT}-Ezrin complex bound to THC. Interaction between Leu542 and Glu207 has the highest occurrence, being present in 39.51% of the simulation frames. **(B)** The graph shows the same residue pair, Leu542 and Glu207, to be interacting most frequently (38.11%) in NSC305787-bound PODXL^{R495W}-Ezrin protein complex. Additional statistics (total interaction, maximum frequency, minimum frequency, and average frequency) are displayed for both complexes.

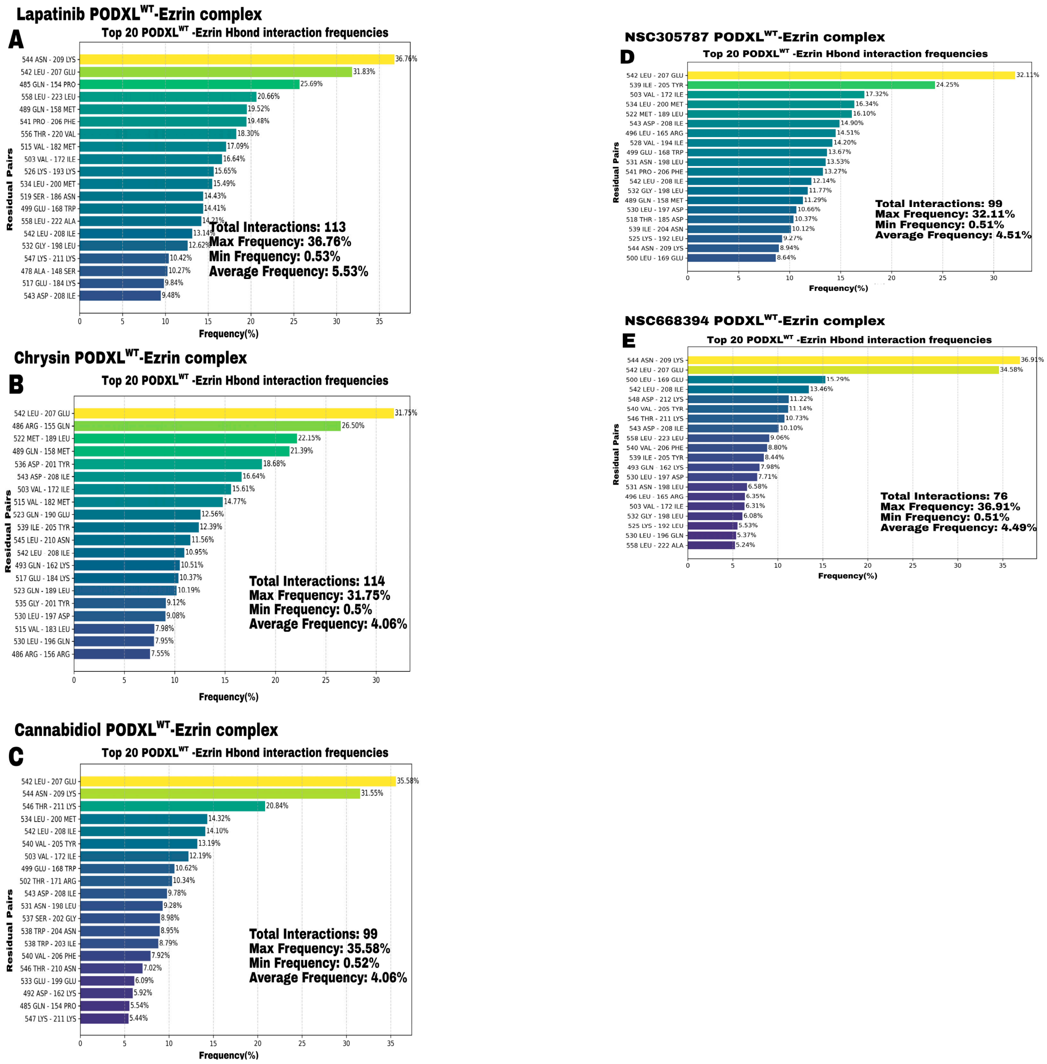

Figure S8. Bar plots showing most frequent hydrogen bond interactions between residues in PODXL^{WT}-Ezrin protein complex upon binding of **(A)** lapatinib, **(B)** chrysin, **(C)** cannabidiol, **(D)** NSC305787, and **(E)** NSC668394. Values for total number of interactions, maximum, minimum, and average frequency are displayed for each complex. **(B)** Chrysin-bound WT complex is the one with the highest number of interactions (114). **(E)** The NSC668394-bound complex exhibits the highest maximum frequency (36.91%).

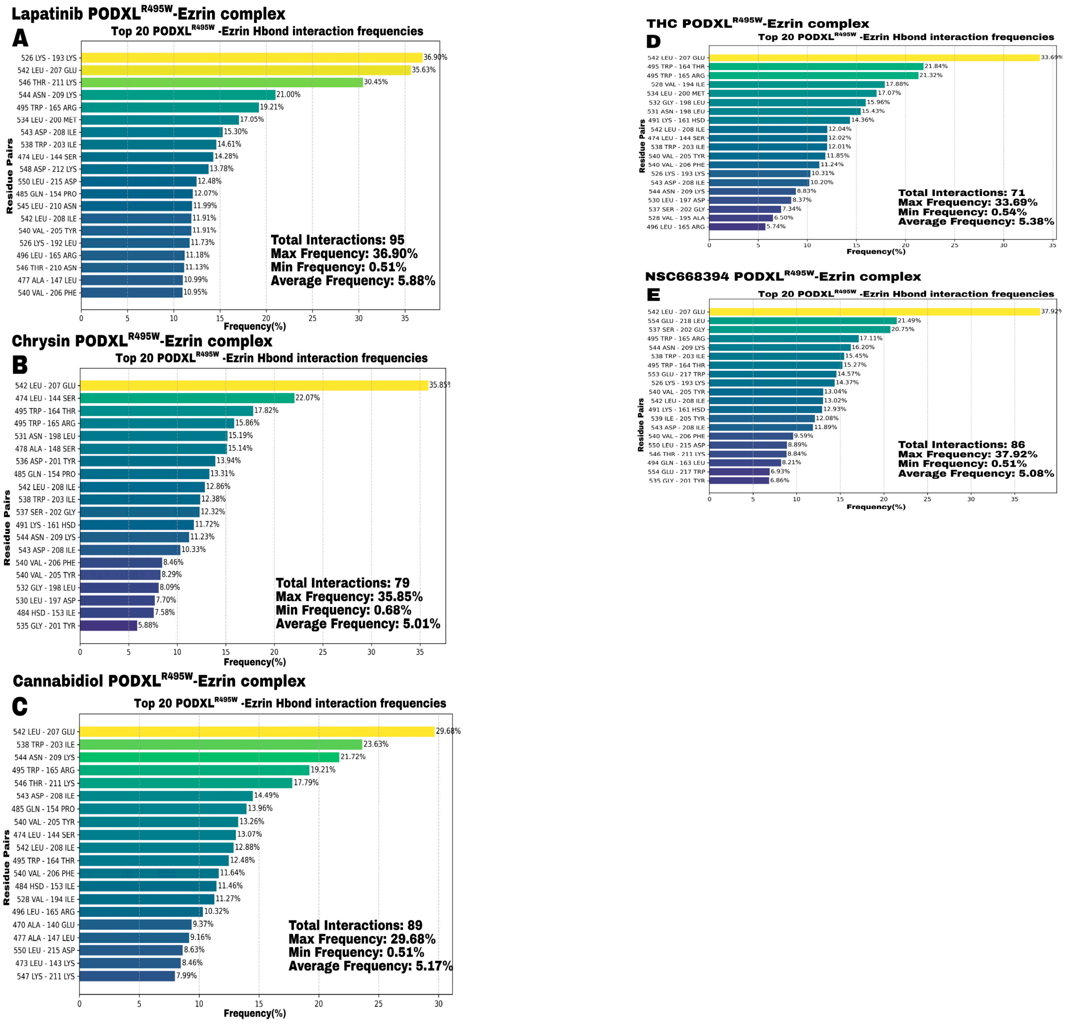

Figure S9. Bar plots showing most frequent hydrogen bond interactions between residues in PODXL^{R495W}-Ezrin protein complex upon binding of **(A)** lapatinib, **(B)** chrysin, **(C)** cannabidiol, **(D)** THC, and **(E)** NSC668394. Values for total number of interactions, maximum, minimum, and average frequency are displayed for each complex. (B) The lapatinib-bound WT complex is the one with the highest number of interactions (95).(E) The NSC668394-bound complex exhibits the highest maximum frequency (37.92%).
